# Vanadium-doped sodium phosphomolybdate salts as catalysts in the terpene alcohols oxidation with hydrogen peroxide†

**DOI:** 10.1039/d1ra04191f

**Published:** 2021-07-08

**Authors:** Castelo Bandane Vilanculo, Márcio José da Silva, Alana Alves Rodrigues, Sukarno Olavo Ferreira, Rene Chagas da Silva

**Affiliations:** Chemistry Department, Pedagogic University of Mozambique, FCNM Campus of Lhanguene, Av. de Moçambique, Km 1 Maputo Zipcode: 4040 Mozambique castelovilanculo@gmail.com +258 825573337; Chemistry Department, Federal University of Viçosa Minas Gerais State 36590-000 Brazil

## Abstract

In this work, we have explored the catalytic activity of Keggin-type heteropolyanions PMo_12−*n*_V_*n*_O_40_^(3+*n*)−^ (*n* = 0, 1, 2, or 3) in the form of sodium salts in green oxidation routes of terpene alcohols with hydrogen peroxide. Nerol was the model molecule selected to assess the impacts of the main reaction parameters, such as temperature, catalyst load, and stoichiometry of reactants. The impacts of the presence of vanadium at different proportions (*i.e.*, V_1_, V_2_, and V_3_ loads/per anion) in the structure of phosphomolybdate catalysts were assessed. All the catalysts were characterized by various techniques such as powder X-ray diffraction, attenuated diffuse reflectance infrared spectroscopy, ultraviolet-visible spectroscopy, thermogravimetric analysis, isotherms of adsorption–desorption of N_2_ measurements of surface area, scanning electronic microscopy, energy-dispersive X-ray spectroscopy, and *n*-butylamine potentiometric titration. Among the catalysts assessed, Na_4_PMo_11_VO_40_ was the most active and selective toward epoxides. The efficiency of this catalyst in the epoxidation of different terpene alcohols was investigated. Special attention was dedicated to correlating the composition and properties of the vanadium-doped phosphomolybdic catalysts with their catalytic activity.

## Abstract 2

In this work, we have explored the catalytic activity of Keggin-type heteropolyanions PMo_12−*n*_V_*n*_O_40_^(3+*n*)−^ (*n* = 0, 1, 2, or 3) in the form of sodium salts in green oxidation routes of terpene alcohols with hydrogen peroxide.

## Graphical abstract

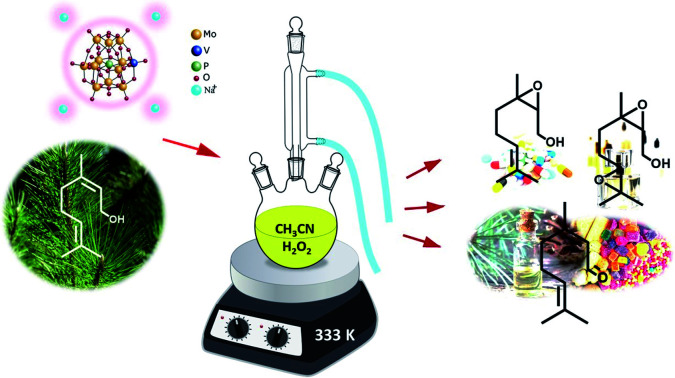

## 1. Introduction

The development of catalysts that can achieve more selective and environmentally friendly oxidation routes of terpenic compounds has received attention due to economic and environmental reasons.^1,2^ Terpenic alcohols are an abundant natural origin feedstock and occur in many plants; they are relevant platform molecules to produce key intermediates for the perfumery, flavoring, fine chemicals, and pharmaceutical industries, being also used as ingredients for the formulation of cosmetics and household products.^3,4^ The oxidation of terpenic alcohol is a synthetic route of interest, leading to the formation of valuable compounds such epoxides, through epoxidation of the olefinic double bond, or carbonylic compounds, after the oxidation of hydroxyl groups.^5,6^ However, most of the oxidative processes that are industrially used still consume hazardous metal stoichiometric oxidants, which should be disposed of into the environment after use.

To address this demand and make the oxidation reactions more benign environmentally, the green and inexpensive oxidants molecular oxygen or hydrogen peroxide have been used in many catalytic processes to oxidize terpenic alcohols, generating water as the only by-product.^7–9^ Nonetheless, it the presence of a metal catalyst is always required to activate these oxidants.^9^ Although molecular oxygen is the most abundant and cheapest oxidant, it is inflammable, and difficult to handle if compared to hydrogen peroxide.^10^ In addition, in certain cases, high pressures of molecular oxygen are sometimes needed for an efficient oxidation.^10^ On the contrary, hydrogen peroxide is a liquid, non-flammable, and an efficient oxidant at room pressure.^11^

Besides the green oxidant, in an oxidative process it is also desirable that the catalyst should be active, selective, and preferentially, easily recoverable.^12^ Different solid catalysts have been widely developed to be used in oxidation reactions of terpenic alcohols.^13,14^ Niobium,^15^ tungsten,^16^ titanium,^17^ and various metal oxides^18,19^ are only some examples of catalysts used in epoxidation reactions of terpenic alcohols with hydrogen peroxide.

Among the different catalysts, Keggin heteropolyacids (HPAs) belonging to the class of polyoxometalates (POMs) have been widely used.^20,21^ These POMs are well-defined metal–oxygen clusters, composed of oxygen atom bridges linking transition metal atoms with high oxidation states, such as vanadium, molybdenum, or tungsten.^22,23^ These versatile compounds have acidity and redox properties that make them efficient catalysts in a plethora of reactions.^24^ Although solid “in nature”, Keggin HPAs have a low surface area and are soluble in polar solvents, hampering their use as heterogeneous catalysts. Therefore, to circumvent this drawback they have been frequently used as solid-supported catalysts.^25–27^

An essential quality of Keggin HPAs consists of their easily adjustable structure. A simple protons exchange by metal cations allows that their properties such as acidity strength, porosity, and surface area can be adequately tuned, making them efficient catalysts in redox or acid-catalyzed reactions.^28–32^ In addition, if the protons are exchanged by large ionic radius cations such as cesium, potassium, or ammonium, they become insoluble catalysts in polar solvents.^33,34^ This modification keeps untouched the Keggin anion, which is the primary structure of these catalysts.

There are still other alterations that allow improving the redox potential of Keggin HPAs; the first one consists of the replacement of one MO unit (*i.e.*, M = Mo, W) generating vacancies into the Keggin anion. Recently, Cs^+^ and Na^+^ cations lacunar HPA salts were successfully used as catalysts in the oxidation of terpenic compounds with hydrogen peroxide.^35–37^ The second modification is to fill the vacancy of lacunar HPA salts with a transition metal cation.^38^ Metal doped phosphotungstic acid salts were effective catalysts in the oxidation reactions of benzylic and terpenic alcohols.^39–41^

Another modification that may improve the activity of Keggin HPAs is to exchange one or more molybdenum atoms by vanadium in the primary structure of catalysts.^42^ Such modification accelerates the steps of oxidation–reduction, which results in the improvement in activity and selectivity in oxidation reactions.^43–46^

An example of improvement triggered by vanadium doping was reported during the isobutane oxidation.^47^ It was described that extra-Keggin O=VO_4_ species attach to the surface of the heteropolyanions, thereby forming V^4+/5+^–O–Mo^6+^ single sites. Although V-free HPAs also catalyze the isobutane oxidation, structures containing vanadium are generally more active. Thus, the aforementioned V^4+/5+^–O–Mo^6+^ single sites are considered the active ones in H_4_PVMo_11_O_40_.^47,48^

Insoluble salts of vanadium-doped phosphomolybdic acid (*i.e.*, Cs^+^ and NH_4_^+^ salts) demonstrated to be active in reactions such as dehydrogenation of propane, oxidation of C_2_–C_4_ hydrocarbons, methacrolein oxidation.^49–51^ Vanadium–molybdenum HPAs have been used as solid-supported catalysts on silica and molecular sieves (*i.e.*, MCM-41, SBA-15), in oxidation reactions of methacrolein and hydrocarbons.^52–54^ Likewise, sodium salts (*i.e.*, Na_5_PMo_10_V_2_O_40_) were supported on active carbon and used in aerobic oxidations.^55^ In a novel approach, H_4_PMo_11_VO_40_ (shell) was supported on hydrothermally treated Cs_4_PMo_11_VO_40_ (core) and successfully used to oxidize methacrolein to methacrylic acid, achieving a higher efficiency than bulky H_4_PMo_11_VO_40_ or H_3_PMo_12_O_40_, or than Cs^+^ salts of these acids.^56^

In this work, we synthesized sodium salts of phosphomolybdic acid, containing Keggin-type heteropolyanions with general formulae PMo_12−*n*_V_*n*_O_40_^(3+*n*)−^ (*n* = 0, 1, 2, or 3), and evaluated their catalytic activity in the oxidation of terpene alcohols with hydrogen peroxide. All compounds were characterized by powder X-ray diffraction, attenuated diffuse reflectance infrared spectroscopy, thermogravimetric analysis, ultraviolet-visible spectroscopy, scanning electronic microscopy, energy-dispersive X-ray spectroscopy, and *n*-butylamine potentiometric titration. The impacts of the main reaction variables were investigated, using nerol as a model molecule. As far as we know, vanadium-containing sodium phosphomolybdates salts were not used yet as catalysts in the oxidation reaction of terpenic alcohols.

## 2. Experimental section

### 2.1. Chemicals

All chemicals were purchased from commercial sources. Nerol, geraniol, and β-citronellol were all Sigma-Aldrich (99 wt%), sodium carbonate (99.5 wt%) and diethyl ether (99.8 wt%) were Proquímicos. Na_2_HPO_4_ (99 wt%) was purchased from Riedel de Hagen. Hydrated heteropolyacid H_3_PMo_12_O_40_ (99 wt%) was acquired from Sigma-Aldrich. V_2_O_5_ (99.6 wt%), MoO_3_ (99.5 wt%), H_3_PO_4_ (85 wt%), NaVO_3_ (98 wt%), Na_2_MoO_4_ (≥98 wt%), CH_3_CN (99 wt%), were also purchased from Sigma-Aldrich. Aqueous hydrogen peroxide (35 wt%) was acquired from Alphatec, and H_2_SO_4_ (95–98 wt%) was Dinâmica.

### 2.2. Synthesis of the Na_4_PMo_11_VO_40_

**Scheme 1:**
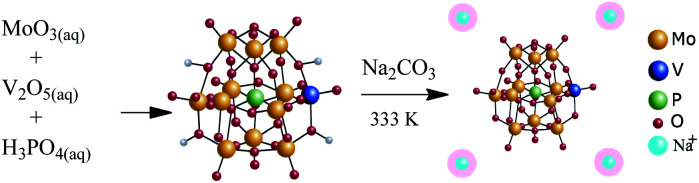
Route of synthesis of Na_4_PMo_11_VO_40_.

The Na_4_PMo_11_VO_40_ salt was synthesized according to the literature.^49^Scheme 1 summarizes the main steps.

Typically, 15.8 g of MoO_3_ and 0.9 g of V_2_O_5_ were dissolved in 350 mL of deionized water and heated to point boiling. Then, 1.2 g of H_3_PO_4_ (85 wt%) was added and the resulting mixture was kept under reflux for 6 h. A clear solution was obtained after cooling to room temperature. The solid acid H_4_PMo_11_VO_40_ was obtained after evaporating the solvent and recrystallization. To synthesize the Na_4_PMo_11_VO_40_ salt, aqueous solutions of Na_2_CO_3_ and H_4_PMo_11_VO_40_ were mixed and heated at 333 K/3 h. Finally, the Na_4_PMo_11_VO_40_ salt was obtained by solvent evaporation, recrystallized from water, and subsequently drying at 373 K/5 h.

### 2.3. Synthesis of the Na_5_PMo_10_V_2_O_40_

**Scheme 2:**
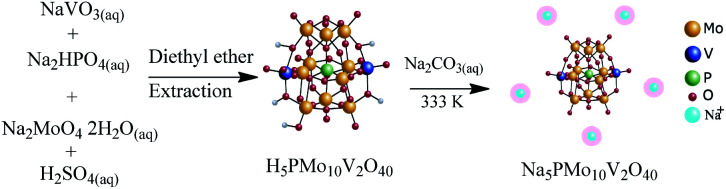
Route of synthesis of Na_5_PMo_10_V_2_O_40_.

The catalyst was synthesized according to the original^55^ and modified procedures^57^ (Scheme 2).

Firstly, an aqueous solution (100 mL) containing Na_2_HPO_4_ (*ca.* 7.1 g) was added to a hot aqueous solution (100 mL) containing 24.4 g of sodium metavanadate. After it has been cooled to room temperature, 5 mL of concentrated H_2_SO_4_ was slowly added, and the solution developed a red color. Afterward, Na_2_MoO_4_·2H_2_O (*ca.* 121 g) was dissolved in water (*ca.* 200 mL) and added to the red solution under vigorous stirring. Concentrated sulfuric acid (*ca.* 85 mL) was slowly added, and the hot solution was cooled to room temperature. Finally, the solution was extracted with ethylic ether, which was vapored under airflow giving the solid acid H_5_PMo_10_V_2_O_40_. The resulting solid was first dried at 343 K, and subsequently dry at 373 K/5 h.

To synthesize Na_5_PMo_10_V_2_O_40_ salt, the aqueous solutions of Na_2_CO_3_ and H_5_PMo_10_V_2_O_40_ were mixed and heated at 333 K/3 hours. The Na_5_PMo_10_V_2_O_40_ salt was obtained by solvent evaporation and recrystallization from water, and dry at 373 K/5 h (Scheme 2).

### 2.4. Synthesis of the Na_6_PMo_9_V_3_O_40_

**Scheme 3:**
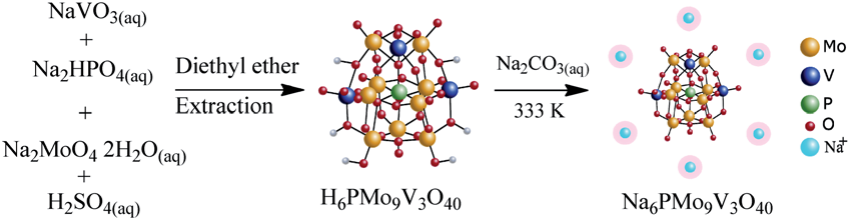
Route of synthesis of Na_6_PMo_9_V_3_O_40_.

A similar route to the described in Section 2.4. was used, except by taking the required amount of sodium metavanadate and sodium molybdate. Herein, the solution resultant developed a cherry red color. After extraction with ethylic ether, it was vapored and recrystallized in water. The Na_6_PMo_9_V_3_O_40_ salt was obtained from H_6_PMo_10_V_3_O_40_ acid following the same procedure used for other sodium salts (Scheme 3).

### 2.5. Characterization of catalysts

Infrared spectra were recorded on a Varian 660-IR spectrometer at a wavenumber range of 400 to 1300 cm^−1^, which is the fingerprint region of the main absorption bands of Keggin heteropolyanions. UV-visible spectra were recorded on a AJX-6100 PC double bean Micronal spectrometer, fitted with tungsten and deuterium lamps to provide visible and UV wavelengths, respectively. The spectra were obtained from CH_3_CN solutions 0.002 mol L^−1^, because it was the concentration used in the most of catalytic runs.

Powder X-ray diffraction patterns of the salts with or without vanadium were analyzed by X-ray diffraction (XRD) spectroscopy using an X-ray Diffraction System Model D8-Discover Bruker using Ni filtered Cu-Kα radiation (*λ* = 1.5418 Å), working at 40 kV and 40 mA, with a counting time 1.0 s in the diffraction angle (2*θ*) ranging from 5 to 80°.

The porosimetry properties of the catalysts were studied by N_2_ adsorption/desorption using NOVA 1200e High Speed, Automated Surface Area, and Pore Size Analyzer Quantachrome Instruments. Before the analyses, the samples were degassed by 1 h. The surface area of the solid catalysts was calculated by the Brunauer–Emmett–Teller (BET) equation, which was applied to the desorption/adsorption isotherms. To characterize the surface of the salts, thin sections were selected and metalized with carbon, for analysis with scanning electron microscopy (SEM) and energy dispersive X-ray spectroscopy (EDS), using a JEOL JSM 6010LA SEM.

Catalyst acidity strength was estimated by potentiometric titration, as described by Pizzio *et al.*^27^ The electrode potential variation was measured with a potentiometer (*i.e.*, Bel, model W3B). Typically, 50 mg of catalyst was dissolved in CH_3_CN and then titrated with *n*-butylamine solution in toluene (*ca.* 0.05 mol L^−1^).

### 2.6. Identification of main reaction products

The main reaction products were identified in a Shimadzu GC-2010 gas chromatograph coupled with a MS-QP 2010 mass spectrometer (*i.e.*, electronic impact 70 eV, scanning range of *m*/*z* 50–450). The purification, spectroscopic characterization, and identification of all the products was previously published.^31,35^

### 2.7. Catalytic runs

Catalytic tests were carried out in a 25 mL three-necked glass flask, fitted with a reflux condenser and sampling system, under magnetic stirrer. Nerol was the model molecule. Typically, nerol (*ca.* 2.75 mmol) and H_2_O_2(aq)_ (*ca.* 34 wt%) were solved in CH_3_CN (*ca.* 10 mL) and heated to 333 K. The addition of catalyst (*ca.* 0.66 mol%) started the reaction.

The runs were monitored during 8 h, periodically collecting aliquots and analyzing them in a GC equipment (Shimadzu 2010, FID), fitted with a Rtx®-Wax, capillary column (30 m length, 0.25 mm i.d., 0.25 mm film thickness). Gas chromatographic conditions were as follows: 80 °C (3 min); heating rate (10 °C min^−1^) until 240 °C. Injector and detector temperatures were 250 °C and 280 °C, respectively.

## 3. Results and discussion

### 3.1. Characterization of catalysts

#### 3.1.1. FT-IR spectroscopy

The primary structure of the Keggin anion of phosphomolybdate catalysts has phosphorus as the central heteroatom, tetrahedrally coordinated to 4 oxygen atoms and surrounded by 12 corner-sharing octahedra, each one containing a Mo atom. There are 4 distinct types of oxygen atoms; the first linking the heteroatom to the Mo atom (O_a_), the other two types of oxygen atoms bridging two transition metal atoms in adjoining octahedra (O_b_ and O_c_), and the last a terminal oxygen atom (O_d_). The vibrations of these chemical bonds generate typical bands in the region 500 to 1200 cm^−1^ of the infrared spectrum, recognized as the fingerprint region. If vanadium atoms replace molybdenum atoms in the Keggin anion, may occur a decrease in the symmetry of the anion, leading to the changes in the bands noticed in the infrared spectrum of salts.^58^

**Fig. 1:**
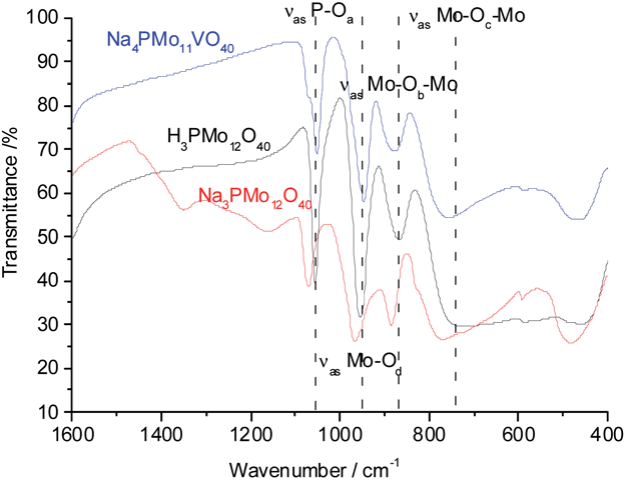
FT-IR/ATR spectra of undoped and doped-vanadium phosphomolybdate sodium salt and phosphomolybdic acid.

To verify if during the synthesis some change occurred in the structure primary (*i.e.*, Keggin heteropolyanion), infrared spectra of phosphomolybdic acid and their undoped and vanadium-doped sodium salt were recorded (Fig. 1).

**Fig. 2:**
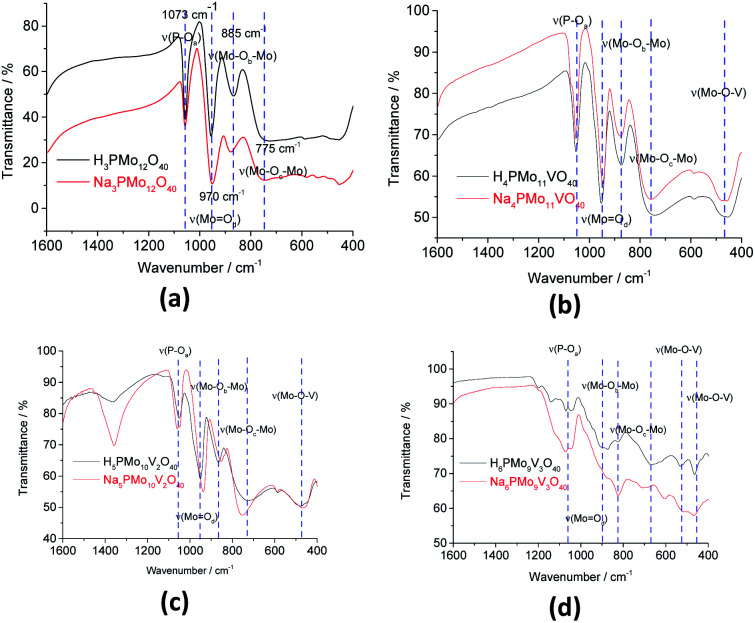
FT-IR spectra of unsubstituted (a) and substituted phosphomolybdic acids (b–d), and their respective sodium salts.

The bands at 1073, 965, 885, and 775 cm^−1^ wavenumbers may be attributed to the asymmetric stretching of the phosphorus–oxygen bond (P–O_a_) of the central tetrahedron (PO_4_), the asymmetric stretching of the bond between molybdenum and different oxygen atoms; peripheral terminal oxygen atom (Mo–O_d_), stretching of the inter-and intra-octahedral bridges of a Mo_3_O_13_ group, Mo–O_b_–Mo and Mo–O_c_–Mo, respectively.^59^ As a reference, dashed lines were inserted in infrared spectra centered at the main absorption bands of phosphomolybdic acids (Fig. 1 and 2). It is possible to note a shoulder in the P–O_a_ bond stretching band (*ca.* 1080 cm^−1^) in the infrared spectrum of the Na_4_PMo_11_VO_40_ salt (Fig. 1). This same effect was previously described in the literature.^60,61^ No significant changes were observed in typical absorption bands of Keggin anion; therefore, it can be concluded that their primary structure was retained after the synthesis of Na_4_PMo_11_VO_40_ salt.

Fig. 2 shows the infrared spectra of undoped and vanadium doped phosphomolybdate acids and their respective sodium salts. A slight shift toward lower frequencies of main absorption bands was also noticed if compared to the infrared spectrum of the Na_3_PMo_12_O_40_ salt.^62^ Moreover, an increase in the replacement level of Mo by V atoms triggered changes in the infrared spectra, which was induced by a decrease in the symmetry of the heteropolyanion.^63,64^ Particularly, the splitting of P–O_a_ bond stretching band (*ca.* 1080 cm^−1^), as well as the presence of shoulders were more noticeable in the vanadium-trisubstituted phosphomolybdate anion.^65^

In the infrared spectra of sodium vanadate–phosphomolybdate salts (Fig. 1SM†), the band corresponding to Mo–O_b_–Mo stretching mode is slightly shifted to lower wavenumber relative to that of Na_3_PMo_12_O_40_. This shift is due to vanadium incorporation into primary structure which forms Mo–O–V linkages by replacing Mo from Mo–O–Mo bonds of Na_3_PMo_12_O_40_.

The shift toward lower frequencies suggests that the strength of the bond decreases when V(v) cations replaced Mo(vi) ions.^66^ The shift is proportional to the number of vanadium atoms in the heteropolyanion. Nevertheless, when it is not observed, possibly may have occurred the elimination of part of vanadium from the primary structure (Keggin) toward the secondary structure during a step of thermal treatment.^67^

**Fig. 3:**
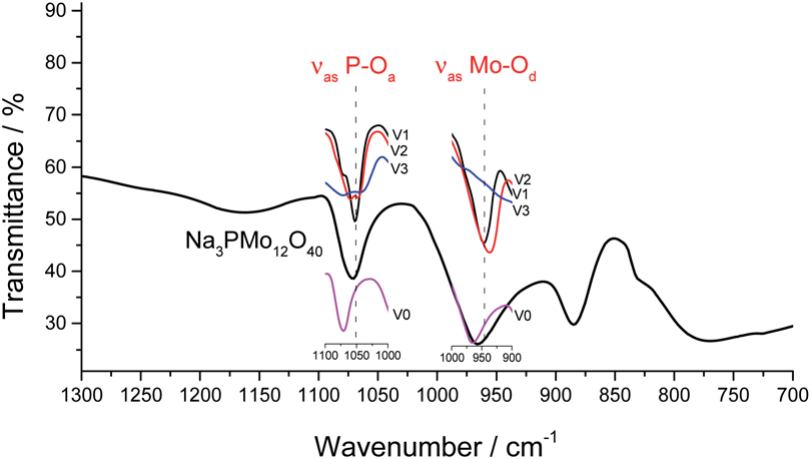
Expanded FT-IR spectra of sodium phosphomolybdate salt and vanadium-substituted sodium phosphomolybdate salts.

It was possible to assume that the Keggin structure of sodium phosphomolybdate salt was retained because no relevant change was noticed in infrared spectra before and after the vanadium doping. The impacts of vanadium load can be clearly seen in the expanded infrared spectra (Fig. 3). Special attention should be devoted to the absorption band assigned to the P–O_a_ bond stretching (*ca.* 1050 cm^−1^) and for the absorption band attributed to the vibration of the Mo–O_d_ bond placed around 950 cm^−1^.

It can be verified that an increase in the number of vanadium atoms led to a clear shift in the position of the bands (*ca.* 1050 cm^−1^ and 950 cm^−1^) toward lower wavenumbers. In addition, the band at 1050 cm^−1^ in the FT-IR spectrum of trisubstituted salt was broadened (Fig. 3). The IR spectra indicate that the structure of the Keggin anion retains upon the molybdenum substitution by vanadium.

#### 3.1.2. UV-visible spectroscopy

The UV-visible spectroscopy in solution is a simple tool to evaluate the potential activity of HPAs as catalysts in the oxidation reactions carried out in the liquid phase. The absorption edge in the UV-visible spectra of HPAs measures the energy necessary for “d–d”-type transitions, which involves the transfer of one electron from the Highest Occupied Molecular Orbital (HOMO) to the Lowest Unoccupied Molecular Orbital (LUMO), or additionally, Ligand to Metal Charge Transfer (LMCT) transitions.^42^

**Fig. 4:**
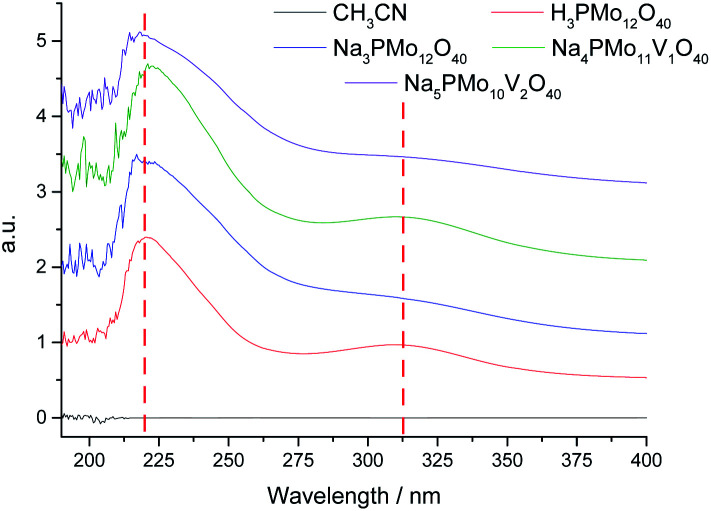
UV-Vis spectra of undoped and doped phosphomolybdic catalysts.

Basically, the HOMO involves mostly the terminal oxygen atoms, consequently, its energy is not remarkably affected by changes in the HPA framework. Conversely, as the LUMO involves the bridging oxygen atoms and the d-orbitals of the metal framework, it can be affected by modifications performed in the heteropolyanion. For instance, the substitution of one or more Mo atoms by V modifies the absorption edge and reflects changes in the LUMO energy, as well as may also impact the redox properties of cluster.^68^ To check how the vanadium doping impacted these transitions, we recorded UV-Vis spectra of the phosphomolybdic catalysts before and after the doping with vanadium atoms (Fig. 4).

The UV-Vis spectra were recorded in acetonitrile solutions. The most intense absorption band was observed at 219 nm wavelength for all the catalysts, which were assigned to the octahedrally coordinated Mo(vi) cations (Fig. 4). A second band was observed at 313 nm but with a weak intensity. The Mo(vi) cations and V(v) have d^0^ configurations, therefore, these bands were attributed to the Mo–O and V–O charge-transfer bands (*i.e.*, LMCT transitions) involving octahedrally coordinated Mo(vi) cations and oxygen atoms.^69^ The vanadium replacement resulted in a shift to red of these absorption band edges to a lower-energy region and the reinforced the absorbance. The doping with vanadium(v) ions led to appearance of multiple small bands at a wavelength lower than 205 nm (*ca.* 190–205 nm), which increased with a higher vanadium doping (Fig. 4). The trisubstituted POMs were insoluble in CH_3_CN and had their UV-Vis spectra obtained in water (Fig. 2SM†). A similar profile was observed for vanadium trisubstituted sodium salt.

#### 3.1.3. Measurement of acidity strength of undoped and vanadium-doped phosphomolybdate catalysts

**Fig. 5:**
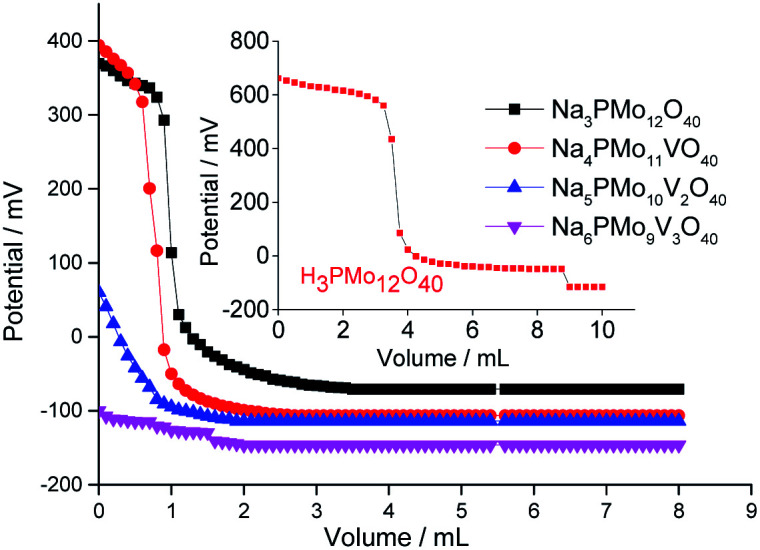
Potentiometric titration curves with *n*-butylamine of pristine phosphomolybdic acid, sodium phosphomolybdate, and vanadium-doped sodium phosphomolybdate salts.

The titration curves of the pristine phosphomolybdic acid, sodium phosphomolybdate, and vanadium-(mono-, di-, or tri-) substituted sodium phosphomolybdate salts are presented in Fig. 5. This technique allows to quantify the total number of acid sites in the catalyst surface, calculated when the plateau of titration curve is reached, and classify the acidity strength, in accordance with the value of the initial potential electrode; *E*_i_ > 100 mV (very strong sites), 0 < *E*_i_ < 100 mV (strong sites), −100 < *E*_i_ < 0 (weak sites) and *E*_i_ < −100 mV (very weak sites).^27^

According to Fig. 5, although have been observed a decrease in the initial potential electrode (*ca. E*_i_ = 680 mV, phosphomolybdic acid; to 400 and 370 mV, unsubstituted and vanadium-monosubstituted sodium salts, respectively), the protons exchange by sodium ions was not enough to diminish the acid strength of the phosphomolybdate catalysts, consequently, both salts presented very strong acid sites (Fig. 5). Indeed, the presence of one vanadium atom had a minimum effect on the acidity strength of sodium salts, and Na_3_PMo_12_O_40_ and Na_4_PMo_11_VO_40_ had titration curves almost similar with the same initial *E*_i_ values. Nonetheless, an increase in vanadium load dramatically reduced the acidity strength of sodium phosphomolybdate salts (Fig. 5).^70,71^

Villabrille *et al.* verified that equally to the observed herein, an increase in vanadium doping, lead to a diminution of the acidity strength of phosphomolybdic acids salts.^72^ They attributed this effect to the increase in charge of heteropolyanion, with a consequent increase in the number of protons, reducing the acidity strength. Although this technique does not distinguish the acid sites (*i.e.*, Lewis or Brønsted), it can be useful to compare the activity of these catalysts in oxidation reactions.^51^

#### 3.1.4. Analyses of powder X-ray diffraction patterns of undoped and vanadium-doped phosphomolybdate catalysts

**Fig. 6:**
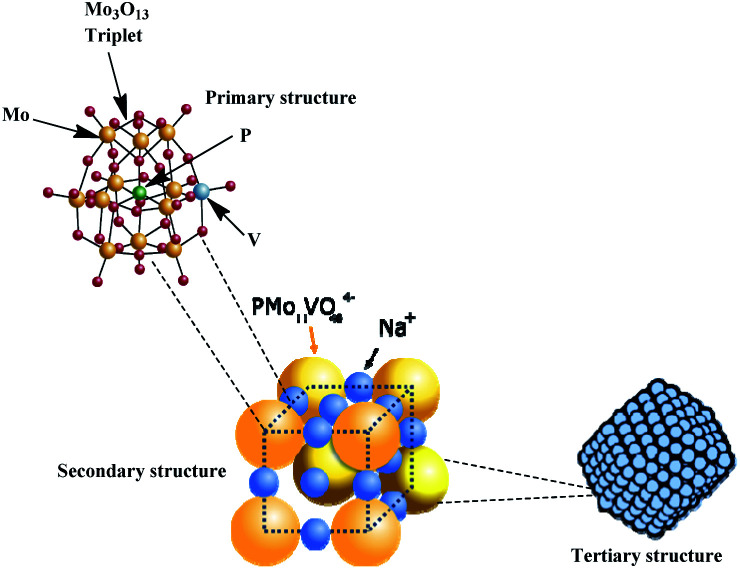
Hierarchical structure of vanadophosphomolybdate sodium salt (adapted from ref. 22).

While infrared spectra of the HPAs offers data about the primary structure of Keggin HPAs (*i.e.*, Keggin heteropolyanion), X-ray spectroscopy is important to assess the secondary structure of these heteropolyanions (Fig. 3SM†). The presence of metal cations and hydration water molecules may affect the arrangement and symmetry of the unitary cell of HPAs.^33,43^ The hierarchical structure of Na_4_PMo_11_VO_40_, which comprises the primary, secondary, and tertiary substructures is showed in Fig. 6.

**Fig. 7:**
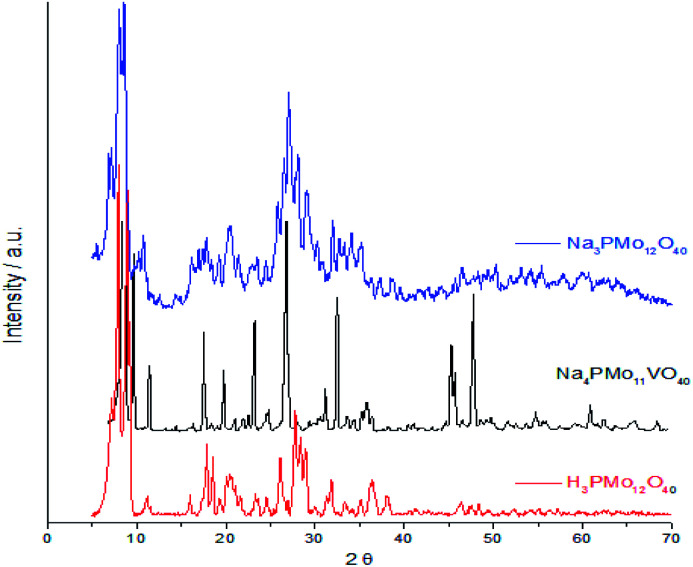
Powder XRD patterns of phosphomolybdic acid before and after the protons exchange by sodium and the vanadium doping.

Fig. 7 displays powder XRD patterns of phosphomolybdic acid before and after the protons exchange by sodium ions and doping of the anion with vanadium. The X-ray diffraction peaks was taken in the 2*θ* ranges of 5.0° to 70.0°. The most significant peaks in the diffractogram of phosphomolybdic acid were observed at 2*θ* values of (5.0–10.0)°, (18.0–23.0)°, and (25.0–30.0)° counts.

The vanadium doping result in a higher crystallinity, preserving the main diffraction peaks between 5 to 40° 2*θ* angle. Nonetheless, there are new diffraction signals at 2*θ* angles greater than 40° (*ca.* 47, 50, and 63° 2*θ* angles), in the diffractogram of the vanadophosphomolybdate sodium salt (Fig. 7).

The literature describes that X-ray diffractograms of the phosphomolybdic acid present well-defined peaks, which suggests that the Keggin anion structure crystallizes in a body-centered cubic system.^73^ Although this pattern depends on the hydration level, the protons exchange is another aspect that also affects the secondary structure. Nonetheless, comparing our data with those reported in the literature we can conclude that the secondary structure remained almost intact after the inclusion of one vanadium atom.^74^

**Fig. 8:**
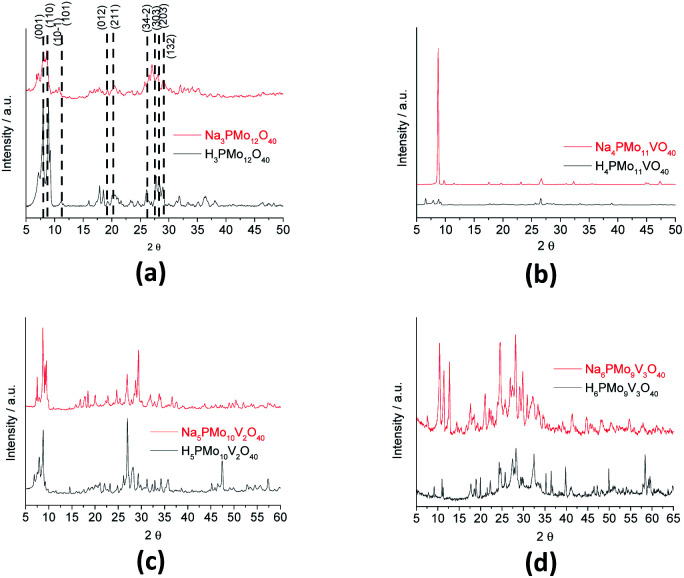
FT-IR spectra of unsubstituted (a) and substituted phosphomolybdic acids (b–d), and their respective sodium salts.

Fig. 8 shows the infrared spectra of undoped and vanadium doped phosphomolybdate acids and their respective sodium salts. In Fig. 8a, were highlighted the diffraction peaks of phosphomolybdic acid which were observed at 2*θ* = 8.1, 8.9, 9.3, 11.1, 11.5, 17.9, 20.1, 26.1, 27.8, 28.3 and 28.9° are respectively attributed to diffraction lines (001), (110), (10−1), (101), (011), (012), (2−1−1), (34−2), (303), (203) and (132).^73^

These values agree with the literature that suggests a cubic crystalline structure for this heteropolyacid.^72,73^ On the other hand, there works that report that H_3_PMo_12_O_40_ and H_4_PVMo_11_O_40_ exhibited similar XRD patterns, consistent with a triclinic lattice, but after exchange protons by Cs^+^ cations, the salts had different crystallinity patterns from the acid forms, associated with a cubic crystallization lattice.^75,76^ Although smaller than Cs^+^ cations, Na^+^ ions are bigger than H^+^ ions and maybe produce the same effect.

**Fig. 9:**
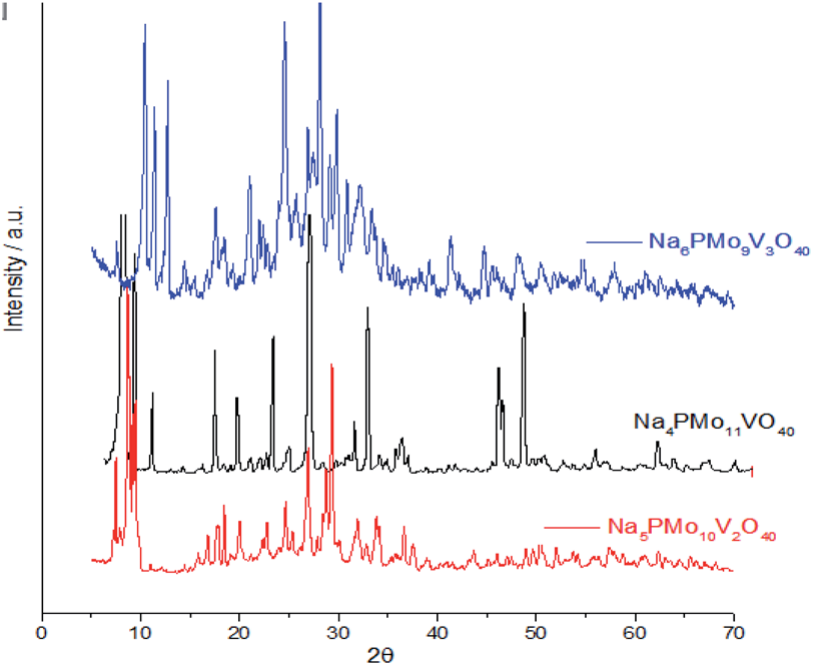
Powder XRD of vanadium (mono, di-, or tri)-substituted sodium phosphomolybdate salts.

Comparing the crystallinity of vanadium doped salts and their acid precursor, we can conclude that they are also highly crystalline, mainly the monosubstituted Na_4_PMo_11_VO_40_ salt, which showed a very intense peak, in the region of 5–10° 2*θ* angle, besides two significant peaks between 45–50° 2*θ* (Fig. 9).

The crystallinity of vanadium doped phosphomolybdic acids and their sodium salts was compared in Fig. 8. Although the sodium has an ionic radium greater than H^+^ ions, the profile of diffractograms was relatively similar. It suggests that both Keggin anion structure and the secondary structure was retained. However, it is required a more deeply study to verify how the vanadium load impacted these structures, mainly when three vanadium atoms are incorporated. It is possible that two types of arrangement had been originated when this modification was performed, generating isomeric Keggin anions.

#### 3.1.5. Thermal analyses of vanadium-containing sodium phosphomolybdate salts

**Fig. 10:**
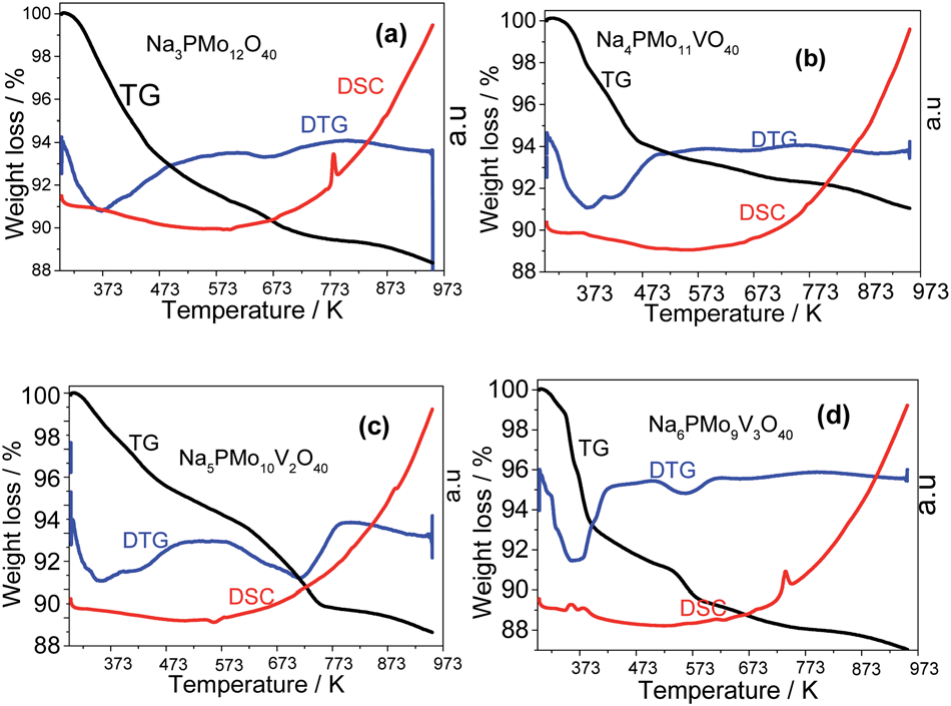
TG-DTG and DSC curves of unsubstituted (a) and vanadium-substituted sodium phosphomolybdate salts (b–d).

TG-DTG and DSC curves obtained from vanadium-containing polyoxometalate salts were obtained under nitrogen atmosphere revealing that two or three thermal events have occurred (Fig. 10). The profile of the TG curve shows a continuous decay of weight in the range of 298 to 973 K, regardless of sodium salt. Nonetheless, it was faster and more pronounced in the thermogram of the Na_6_PMo_9_V_3_O_40_.

**Scheme 4:**
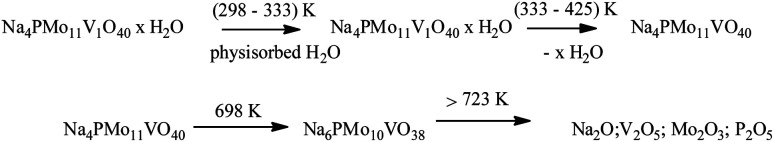
Sequence of thermal events proposed as the basis of weight loss (adapted from ref. 47).

The first weight loss occurred heating the sample from room temperature up to 388 K, with a minimum in the DTG curve at approximately 373 K. It was assigned to the release of physically adsorbed water and some of the coordinate hydrating water molecules. The second weight loss happened in the temperature range from 338 to 572 K and resulted in the formation of anhydrous catalyst. The third event occurred over a wide temperature range (*ca.* 653–803 K), and it was ascribed to the decomposition of Keggin anion and total formation of oxides mixture. This sequence of events was described by Jing *et al.* after they perform the thermal analysis of Keggin salts containing hydrate PMo_11_VO_40_^3−^ anion (Scheme 4).^49^

All the DTG curves had a decline at 473 K temperature, however, while the curves obtained from the samples of Na_3_PMo_12_O_40_ and Na_4_PMo_10_V_1_O_40_ remained almost constant after this temperature (Fig. 10a and b), DTG curves of Na_5_PMo_10_V_2_O_40_ and Na_6_PMo_9_V_3_O_40_ presented minimum at 723 and 573 K, respectively (Fig. 10c and d). The literature describes that Keggin heteropolyanions are decomposed to oxides at temperatures equal or higher than 773 K, a resulted confirmed by the appearance of an endothermic peak in DSC curves.^50,76^ This peak was more visible in the DSC curves obtained from Na_3_PMo_12_O_40_ and Na_6_PMo_9_V_3_O_4_, where it was observed at temperatures of 773 and 743 K, respectively (Fig. 10a and d).

From Fig. 10, it was possible to calculate the number of water molecules present in the salts, considering the percentage of mass loss that occurred in the range of 298 to 523 K, which is assigned to the loss of physisorbed, bounded, and structurally coordinated water molecules.^77^ According to the weight loss percentage, it was possible to quantify the hydration water molecules present in the sodium salts (Table 2SM†). It was verified that as greater the number of Vanadium atoms, higher was the number of water molecules (*ca.* 7, 10 and, 13 water moles, in V1, V2, and V3 catalysts). Some of the changes observed in XRD patterns of sodium salts (Fig. 8 and 9) can be ascribed to the different hydration levels.^78^

#### 3.1.6. Analyses of porosimetry of undoped and vanadium-doped sodium phosphomolybdate salts

The N_2_ adsorption/desorption isotherms provided information about the surface area (BET), volume, distribution, and pores diameter of molybdate sodium salt catalysts (Fig. 4SM†). According to IUPAC rules, the isotherms were classified as being intermediate between type III and V. The same way, the slight hysteresis loops in the isotherm plots were classified as H-3. The vanadium-doped phosphomolybdate salts were classified as a mesoporous material (*ca.* pores size between 5 to 50 nm), with a higher range of pore size when the vanadium doping was increased. It was ascribed to the capillarity condensation in mesopores of the solid catalysts, a consequence of adsorption on aggregates of platy particles.

Keggin HPAs are solid with a low surface area (*ca.* < 5 m^2^ g^−1^), which can be increased if the protons are exchanged by cations with an ionic radius higher than 1.3 Å.^79^ Besides that, depending on the sort of cation, the salts can be insoluble in a polar solvent. In this work, our intention was to assess the activity of the vanadium salts dissolved in the solution, due to this reason, small ions likewise sodium were selected to replace the protons. Therefore, the increase in the surface area was not very pronounced. However, an increase in vanadium load seems to have also contributed to increasing the surface area of salts (Table 1SM†).

#### 3.1.7. SEM-EDS analyses of vanadium-containing heteropoly acid and its sodium salt

The vanadium-containing sodium phosphomolybdate salts were submitted to SEM-EDS analysis to characterize their surface. Fig. 5SM† shows SEM images of the pure sodium phosphomolybdate salt and with different loads of vanadium.

Comparing the SEM images of the vanadium-containing heteropoly salt with the precursor heteropoly acid, it was possible to note that there was a reduction in the particle sizes. Specifically, the particles of the Na_4_PMo_11_VO_40_ are noticeably smaller than those of the undoped Na_3_PMo_12_O_40_ and, therefore, apparently, the insertion of vanadium in the structure of anion increased the surface area, as demonstrated by BET analysis.

The percentual elemental composition of sodium phosphomolybdate salts was confirmed by EDS analysis, which agrees with the theoretical values (Fig. 6SM†). No residual element was found in the samples (*i.e.*, the sulfuric acid component used in the synthesis), a guarantee that they were adequately purified.

### 3.2. Catalytic tests

#### 3.2.1. Effect of the catalyst on nerol oxidation by hydrogen peroxide

**Fig. 11:**
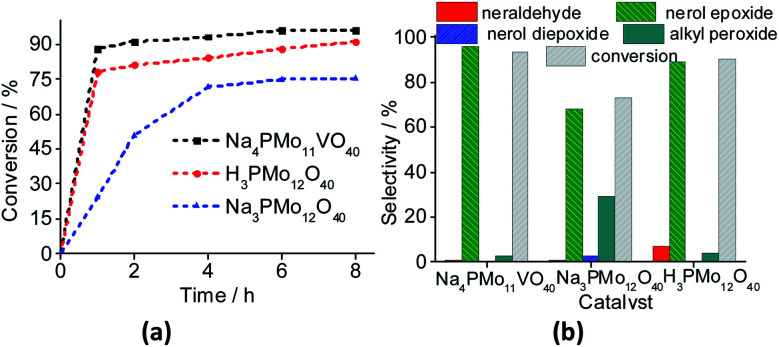
Effect of catalyst nature on kinetic curves (a), conversion and products selectivity after 8 h (b) of nerol oxidation reactions by H_2_O_2_. Reaction conditions: nerol (2.75 mmol), H_2_O_2_ (2.75 mmol), toluene (internal stander), catalyst (0.66 mol%), reaction time (8 h), temperature (333 K), CH_3_CN (10 mL).

In the initial catalytic tests, we have studied the activity of pristine phosphomolybdic acid, and their undoped and vanadium-doped sodium salts in the oxidation reactions of nerol, the selected model molecule (Fig. 11). The reaction conditions were chosen based on the literature.^35,36^

**Scheme 5:**
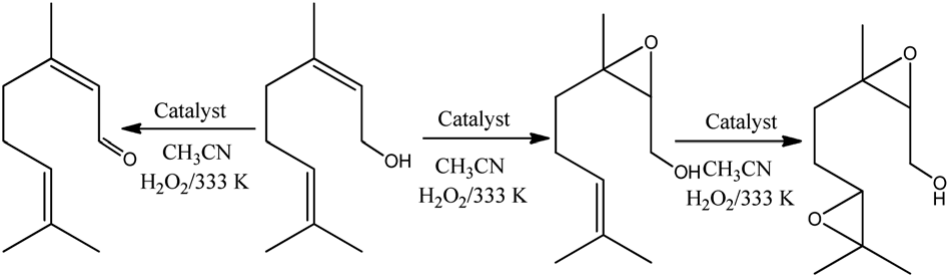
Oxidation of nerol with H_2_O_2_ in the presence of phosphomolybdate catalysts in CH_3_CN solutions.

The reaction conversions were Na_4_PMo_11_VO_40_ > H_3_PMo_12_O_40_ > Na_3_PMo_12_O_40_, evidence that the vanadium doping had a beneficial effect on the performance of sodium phosphomolybdate catalyst (Fig. 11a). Besides the highest initial rate and conversion, this catalyst provided the greatest selectivity toward oxidation products, being the nerol epoxide the major product (Fig. 11b). Nerol diepoxide and neraldehyde were the secondary products (Scheme 5).

The undoped catalyst achieved the lowest conversion (*ca.* 73%), it presented the lowest selectivity toward nerol epoxide. Moreover, it led to the highest formation of alkyl peroxides, possible reaction intermediates, which are non-detected products by GC FID analysis but were calculated through the mass balance of reactions (Fig. 11b).

**Fig. 12:**
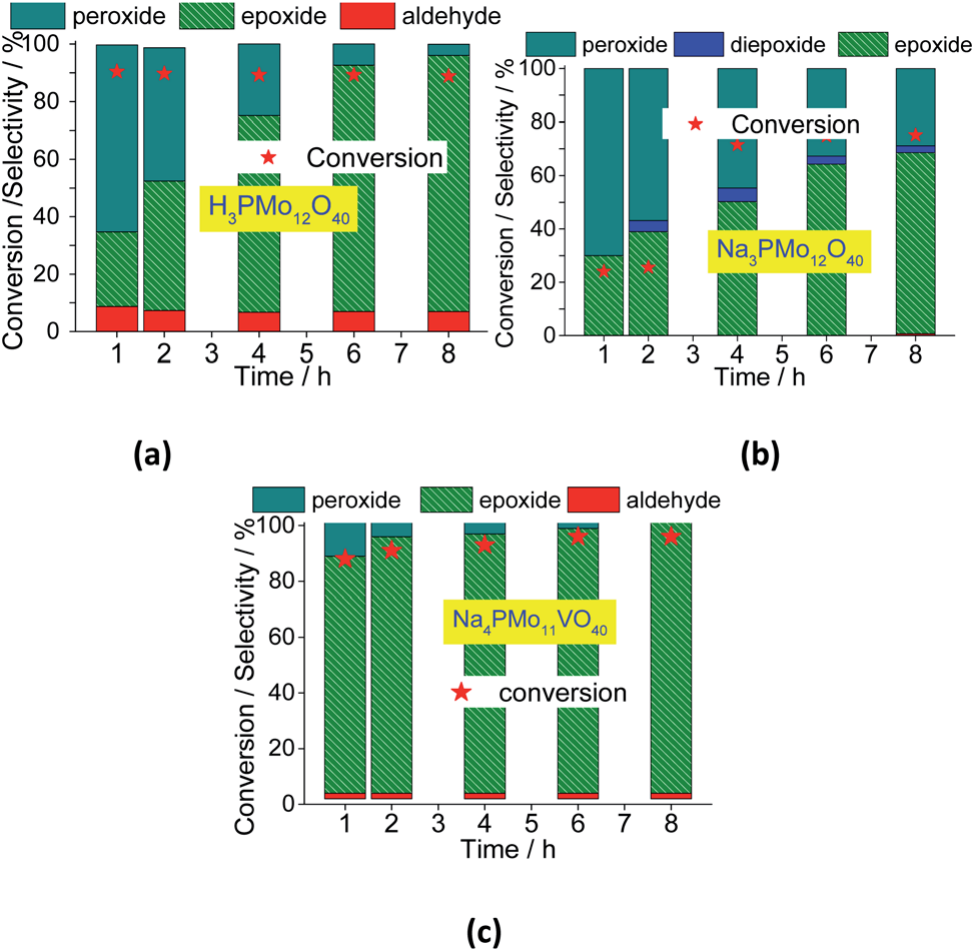
Effect of the catalyst on the conversion and reaction selectivity *versus* time of oxidation reactions of nerol with H_2_O_2_ in presence of H_3_PMo_12_O_40_ (a), Na_3_PMo_12_O_40_ (b) and Na_4_PMo_12_VO_40_ (c) catalysts.

In Fig. 12, it was described how the reaction selectivity and the conversion varied during all the time. Fig. 12a and c show that although the H_3_PMo_12_O_40_ or Na_4_PMo_11_VO_40_-catalyzed reactions had achieved the maximum conversion within the first hour of run, the selectivity of each process progressed differently through the time; while in reaction with Na_4_PMo_11_VO_40_ the nerol epoxide was always the major product, in the presence of H_3_PMo_12_O_40_ catalyst until 4 h reaction it happened with another compound (*i.e.*, nerol peroxide). Only from this time, nerol epoxide became the main product.

Comparing the performance of the two most efficient catalysts, it can be concluded that both Brønsted (*i.e.*, H_3_O^+^ ions, H_3_PMo_12_O_40_), and Lewis sites (*i.e.*, vanadium sites, Na_4_PMo_11_VO_40_), seem to play a crucial role in the conversion of nerol to the epoxide. However, Na_4_PMo_11_VO_40_-catalyzed reaction converted more quickly the reaction intermediates (*i.e.*, nerol peroxide) to the final product (*i.e.*, nerol epoxide) (Fig. 12a and c) than acid-catalyzed reaction. Conversely, the undoped salt (*i.e.*, Na_3_PMo_12_O_40_) was the less efficient catalyst, and its reaction achieved the lowest conversion and nerol epoxide selectivity, leaving consequently the highest amount of nerol peroxide without being converted to the final product (Fig. 12b).

#### 3.2.2. Effect of vanadium load on nerol oxidation by hydrogen peroxide

**Fig. 13:**
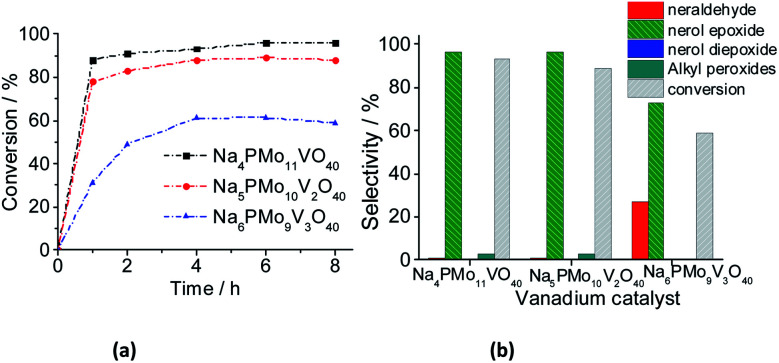
Effect of vanadium doping on the activity of sodium phosphomolybdate catalysts; kinetic curves (a), conversion, and products selectivity after 8 h (b) of nerol oxidation with H_2_O_2_. Reaction conditions: nerol (2.75 mmol), H_2_O_2_ (2.75 mmol), toluene (internal stander), catalyst (0.66 mol%), temperature (333 K), CH_3_CN (10 mL).

When one vanadium atom was introduced in the Keggin phosphomolybdate anion, there was a significant enhancement in the conversion and selectivity toward epoxide of oxidation reaction of nerol (Fig. 12). To verify if this effect is proportional to the vanadium atoms load present in the catalyst, tests were performed with the three vanadium salts (Fig. 13).

When the content of vanadium was increased from V1 to V2, only a slight decrease in the conversion and selectivity was noticed (Fig. 13a and b). Nonetheless, when the sodium phosphomolybdate containing three vanadium atoms was the catalyst, the reaction conversion was remarkably lower (*ca.* 58%), although there was a significant increase in the selectivity toward neraldehyde (*ca.* 27%, Fig. 13b).

A similar effect was noticed when TiO_2_-supported vanadium-doped cesium phosphomolybdate (*i.e.*, Cs_3+*n*_PMo_12−*n*_V_*n*_O_40_/TiO_2_, *n* = 0–3) catalysts were used in the benzyl alcohol oxidation to benzaldehyde.^80^ Despite the different oxidant (*i.e.*, molecular oxygen) has been used, those authors verified that while the Cs_4_PMo_11_VO_40_/TiO_2_-catalyzed reactions reached the highest conversion, an increase in vanadium content led to a decreasing in conversion. Moreover, likewise verified herein, the aldehyde selectivity was also increased. Those authors ascribed this effect to a change in redox properties (*i.e.*, the formation of V^4+^/V^5+^ pairs), and the increase in protons mobility triggered by the increase of the vanadium doping.^80^

**Fig. 14:**
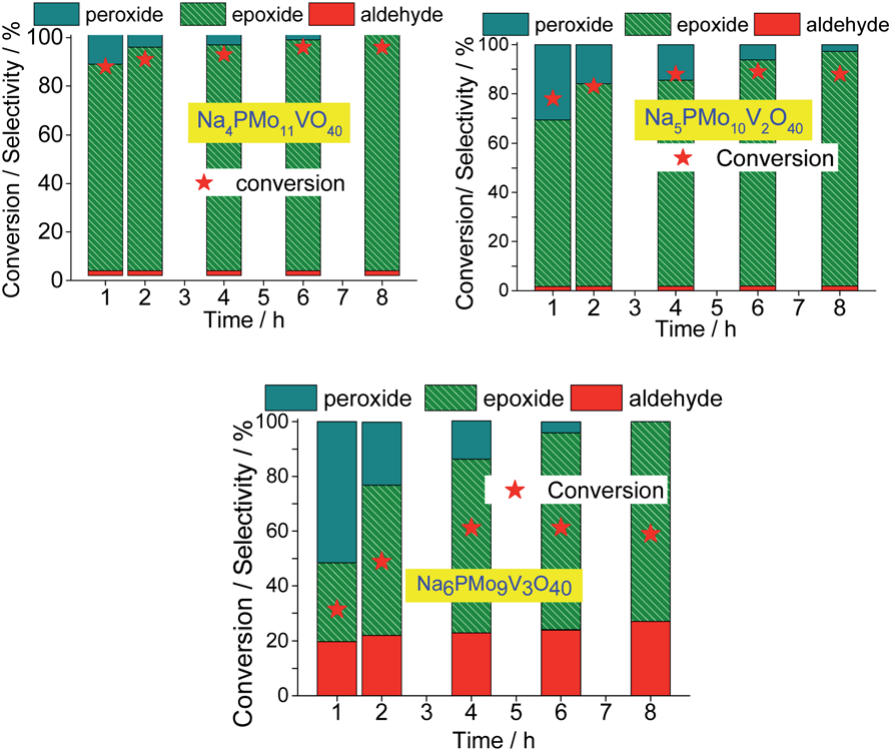
Effect of vanadium doping on the conversion and reaction selectivity *versus* time of Na_3+*n*_PMo_11_V_*n*_O_40_^(3+*n*)−^ (*n* = 1–3) catalyzed oxidation reactions of nerol with H_2_O_2_.

The decline in the performance of the catalyst observed when a higher vanadium load was used can be justified by the increase of energy barrier between HOMO and LUMO orbitals, which difficult the reducibility of these di-or tri-substituted heteropolyanions.^80^ The evolution of reaction selectivity along the process was also monitored and the main data area shown in Fig. 14.

The selectivity of oxidation products reached in the vanadium salts-catalyzed reactions (*i.e.*, mainly nerol epoxide and neraldehyde) was as follows: Na_4_PMo_11_V_1_O_40_ > Na_5_PMo_10_V_2_O_40_ > Na_6_PMo_9_V_3_O_40_, the same trends observed in terms of conversion.

When we focus on the selectivity of nerol peroxide, we can realize that as higher was the reaction conversion, more quickly the nerol peroxide was converted to oxidation products. It means that in the first hour of reaction, the highest selectivity of nerol peroxide was found in the reaction with the less efficient catalyst (*i.e.*, Na_6_PMo_9_V_3_O_40_). Once more, it can be attributed to the higher content of vanadium. The doping of phosphomolybdate anion with three vanadium atoms drastically reduces the acidity strength of the catalyst (Fig. 5), compromising its performance. An aspect that distinguishes this reaction from the others is the significant formation of neraldehyde. Nonetheless, since the epoxidation reactions oxidation of alcohol to aldehyde involves different mechanisms, we will discuss them in the next section.

#### 3.2.3. Mechanistic discussions

It is noteworthy that epoxidation of allylic alcohols with hydrogen peroxide in the presence of metal catalysts such as tungsten, titanium, molybdenum, or niobium are hydroxyl group assisted-reactions.^15–17,37,81–83^ This reaction competes with the oxidation of alcohol to aldehyde, and the chemoselectivity will depending on the coordinate fashion of substrate to the metal catalyst.

**Scheme 6:**
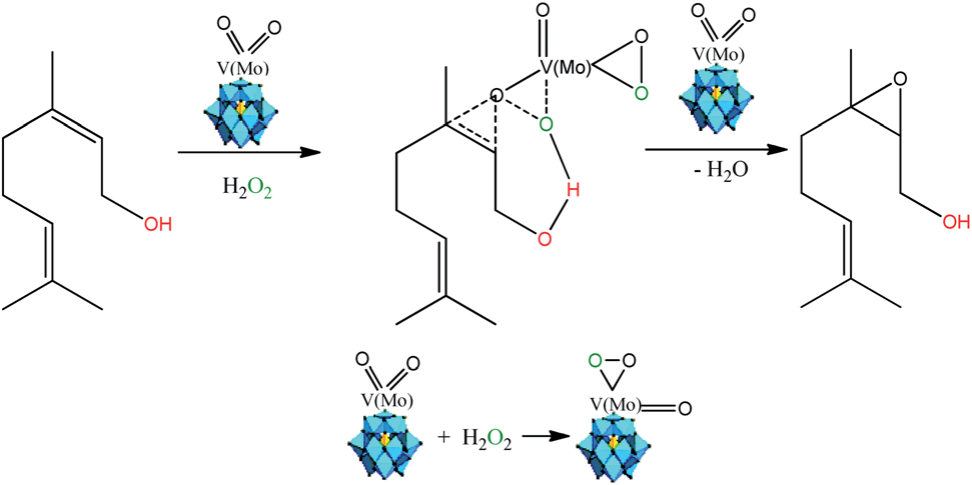
Propose of reaction pathway of vanadium-doped sodium phosphomolybdate salt-catalyzed nerol epoxidation reaction with hydrogen peroxide (adapted from ref. 15, 17 and 38).

Several authors have described that when Keggin HPAs are the catalysts in oxidation with peroxide hydrogen of alcohols or olefins, peroxidized intermediates are the most probable active species.^15,17,37,84^ While in the epoxidation of alkenes with hydrogen peroxide in the presence Ti-containing HPA catalysts the hydroperoxide species (*i.e.*, –OOH) are the most common intermediates, in those with vanadium, molybdenum, or tungsten the oxo-intermediates (*i.e.* –O–O– species) are the most favored.^85,86^ Therefore, as the basis of the literature and our experimental results we proposed that nerol epoxidation maybe describes as in Scheme 6.^15,38^

Herein, we suppose that the epoxidation is also a hydroxy group assisted-reaction that involves a peroxidized vanadium-doped phosphomolybdate intermediate (Scheme 6). Although molybdenum atoms are also the active sites, our results showed that the formation of nerol epoxide was more favorable when one vanadium atom were present in the Keggin anion. Indeed, Romanelli *et al.* described that the formation of the peroxide–metal intermediate between the peroxide and the HPA catalyst (*i.e.*, Mo or W addenda atoms) is more favorable in the presence of vanadium atom.^87,88^ This beneficial effect was ascribed to the reduction of the energy gap between HOMO and LUMO orbitals, provoked by the presence of one vanadium atom, which favors the reducibility of Keggin anions, as demonstrated in studies of molecular orbital performed with vanadium-doped phosphomolybdic acids.^89^

It is important to highlight that even in the absence of vanadium, the reaction efficiently proceeded; indeed, it was more visible when a strong Brønsted acidity strength was present, which is translated into better catalytic performance, as in the case of H_3_PMo_12_O_40_-catalyzed reaction.^90^ As demonstrated the potentiometric titration curves, H_3_PMo_12_O_40_ and Na_4_PMo_11_VO_40_ presented high values of initial electrode potential, confirming that they have very strong acid sites (Fig. 5).^27^

The H_3_PMo_12_O_40_ catalyst in the solid-state present different protons in their structure (*i.e.*, H^+^, H_3_O^+^, and H_5_O_2_^+^).^90,91^ We believe that in solution, the less mobile protons linked to the Keggin anion can interact with hydrogen peroxide through intermolecular hydrogen bonds. This effect favors the peroxidation of the phosphomolybdate anion, which favor the formation of an oxo-intermediate, likewise to the depicted in Scheme 6, however, having Mo as the atom present in oxo-moiety. Consequently, this intermediate can transfer an oxygen atom to the nerol double bond. It is noteworthy that even with a low concentration of protons (*i.e.*, Na_3_PMo_12_O_40_), which has only residual protons, the oxidation reaction was less efficient (Fig. 13). Conversely, with a typically protic catalyst (*i.e.*, H_3_PMo_12_O_40_), the oxidation reaction reached a high conversion and selectivity toward nerol epoxide (Fig. 11).

**Scheme 7:**
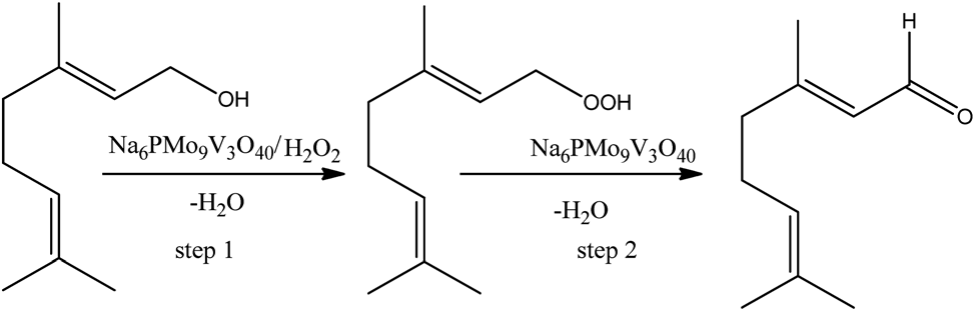
Propose of reaction pathway of Na_6_PMo_9_V_3_O_40_-catalyzed nerol oxidation reaction with hydrogen peroxide.

On the other hand, the highest selectivity to neraldehyde was achieved in the Na_6_PMo_9_V_3_O_40_-catalyzed reaction. In general, such reactions can be explained as in Scheme 7, where the hydroxyl group of alcohol is peroxidized, and further this intermediate is decomposed by the catalyst to neraldehyde and water.^91^

Although the peroxidation of an alcohol can occurs without a catalyst, we suppose that herein it participates either in the transference step of an oxygen atom from oxidant to alcohol (step 1, Scheme 4), as in the decomposition of the peroxide intermediate to aldehyde (step 2, Scheme 7). To do it, it is requiring that the catalyst should be easily oxidizable, it is, undergone a fast interconversion of one electron (*i.e.*, V^4+^/V^5+^).^92^ The literature suggests that a high load of vanadium can promote this process.^89–92^ Another important aspect is linked to the acidity of the catalyst. This catalyst was who presented the lowest acidity strength (Fig. 5). Previously, it has been reported that in Nb_2_O_5_-catalyzed nerol oxidation reactions the niobium oxide with the weakest acid sites proved the highest neraldehyde selectivity.^15^

#### 3.2.4. Effect of the load of Na_4_PMo_11_VO_40_ catalyst on nerol oxidation by H_2_O_2_

**Fig. 15:**
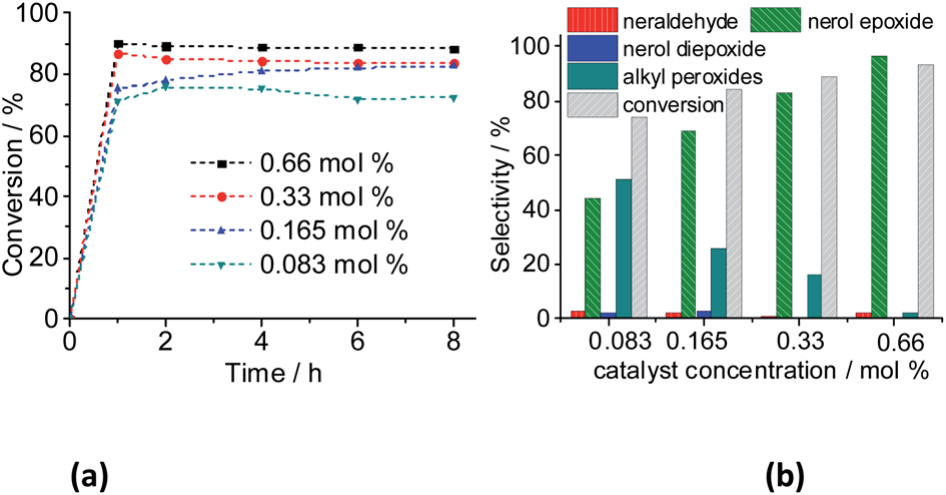
Impacts of Na_4_PMo_11_VO_40_ catalyst load on the kinetic curves (a), conversion, and products selectivity after 8 h (b) of nerol oxidation reactions with H_2_O_2_. Reaction conditions: nerol (2.75 mmol), H_2_O_2_ (2.75 mmol), toluene (internal stander), temperature (333 K), CH_3_CN (10 mL).

The catalytic activity of Na_4_PMo_11_VO_40_ was evaluated using different concentrations, and the main results are displayed in Fig. 15. An increase in catalyst load enhanced the initial rate of reactions, nonetheless, the kinetic curves had the same profile, achieving the maximum conversion within the initial period, regardless of the catalyst load.

**Fig. 16:**
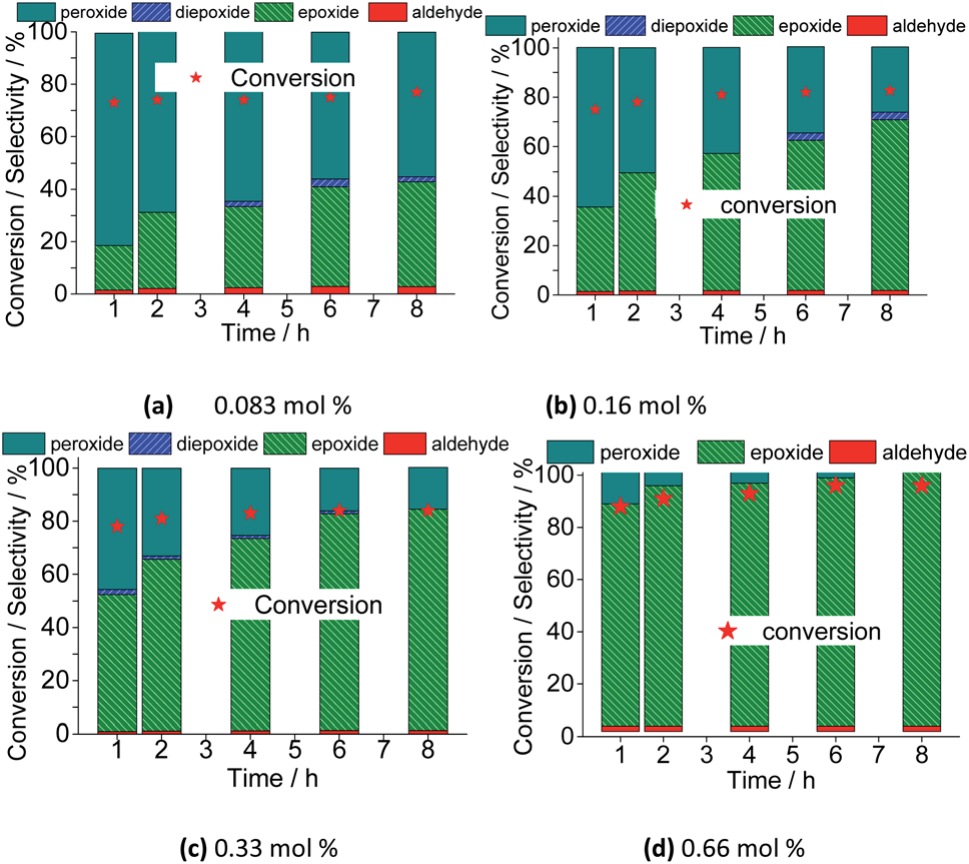
Variation of conversion and reaction selectivity *versus* time of Na_4_PMo_11_VO_40_-catalyzed nerol oxidation reaction with H_2_O_2_.

However, the selectivity had a different behavior; within the first hour of the reaction, the nerol was quickly converted to oxidation products, with a great quantity of alkyl peroxides, which were being gradually converted to nerol epoxide while the reaction proceeds. Therefore, although the maximum conversion has been quickly reached, it is required to carry the reaction by longer times. Moreover, the decomposition of alkyl peroxides to oxidation product (*i.e.*, nerol epoxide) was more efficient if a higher catalyst load was present in the solution. This effect can be visualized in Fig. 16, which shows the conversion and selectivity of reactions along the time.

Regardless of the catalyst load, all the runs achieved the maximum conversion within 2 h of reaction, having always nerol epoxide as the main product (Fig. 16).

#### 3.2.5. Effect of oxidant load on the conversion and selectivity Na_4_PMo_11_VO_40_-catalyzed oxidation with H_2_O_2_

**Fig. 17:**
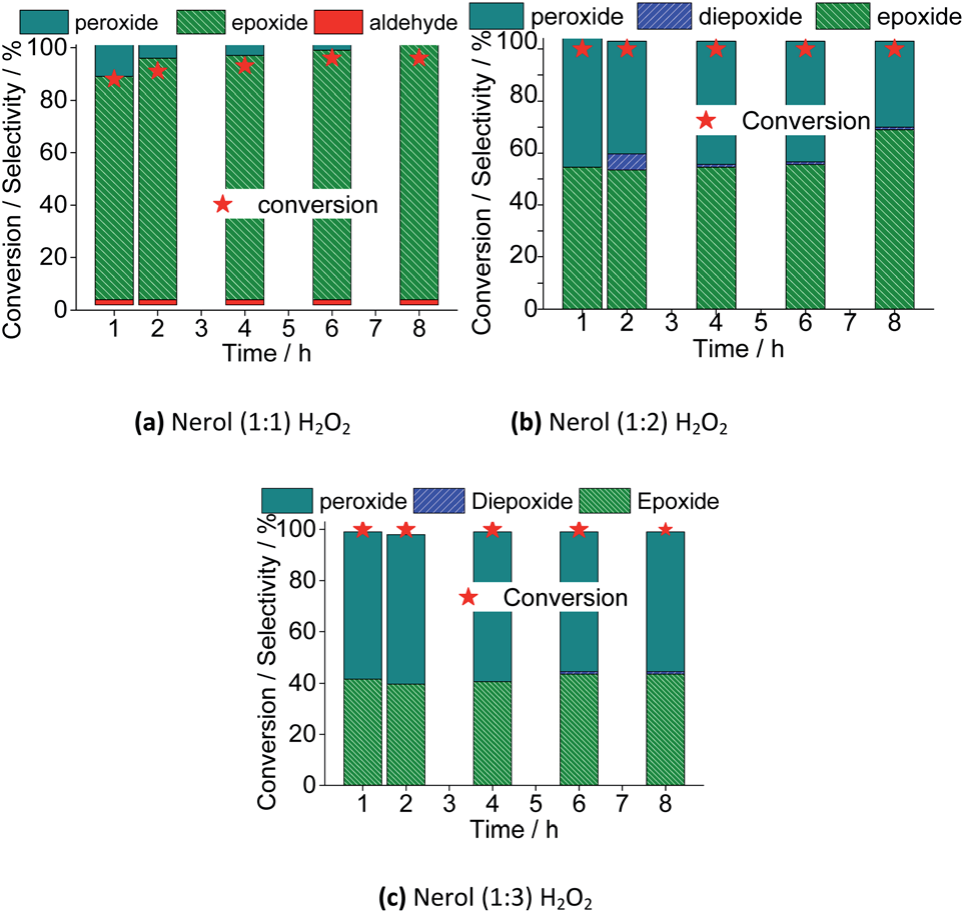
Effect of stoichiometry of the reactants on the conversion and reaction selectivity *versus* time of Na_4_PMo_11_VO_40_-catalyzed oxidation reactions of nerol with H_2_O_2_. Reaction conditions: nerol (2.75 mmol), Na_4_PMo_11_VO_40_ (0.66 mol%), temperature (333 K), CH_3_CN (10 mL).

As we reported in our previous work,^15,17^ the amount of the oxidant could affect the substrate conversion and the product's selectivity, mainly due to the presence of greater water amount when a greater excess of oxidant is used. This effect was evaluated the main results are in Fig. 17.

The excess of peroxide had different impacts on reaction rate and selectivity of products. Regardless of the oxidant load, the maximum conversion was achieved within the first hour of reaction. Afterward, the conversion remained unaltered. Nonetheless, keeping constant the catalyst load, a greater amount of oxidant has no positive effect in converting the alkyl peroxides to nerol epoxide. It suggests that a large excess of peroxide compromises the formation of active species from the peroxidized catalyst and the substrate. Possibly, the alcohol itself is peroxidized, and the interaction of these species and the oxo-catalyst its less favorable. Verifying the conversions in uncatalyzed reactions, we have found that using 1 : 1, 1 : 2, and 1 : 3 molar ratios, conversions of <5, 10, and 15% were reached, however, no significant amount of oxidation products was detected. It assures that the substrate is peroxidized (*i.e.*, once that it is converted), however, nerol epoxide and neraldehyde were not detected.

#### 3.2.6. Assessment of the effect of temperature on the Na_4_PMo_11_VO_40_-catalyzed oxidation of nerol by H_2_O_2_

**Fig. 18:**
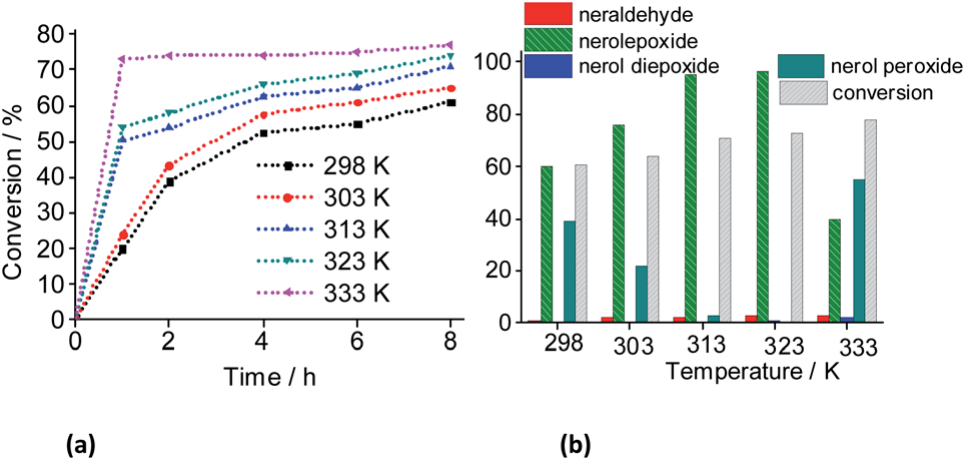
Influence of temperature on the kinetic curves (a), conversion and products selectivity after 8 h of (b) Na_4_PMo_11_VO_40_-catalyzed oxidation reactions of nerol with H_2_O_2_. Reaction conditions: nerol (2.75 mmol), H_2_O_2_ (2.75 mmol), Na_4_PMo_11_VO_40_ (0.083 mol%), CH_3_CN (10 mL).

To investigate the influence of the temperature on the catalytic performance of the Na_4_PMo_11_VO_40_ salt, we have used a low catalyst concentration (*ca.* 0.083 mol%), to do a more visible this effect (Fig. 18).

**Fig. 19:**
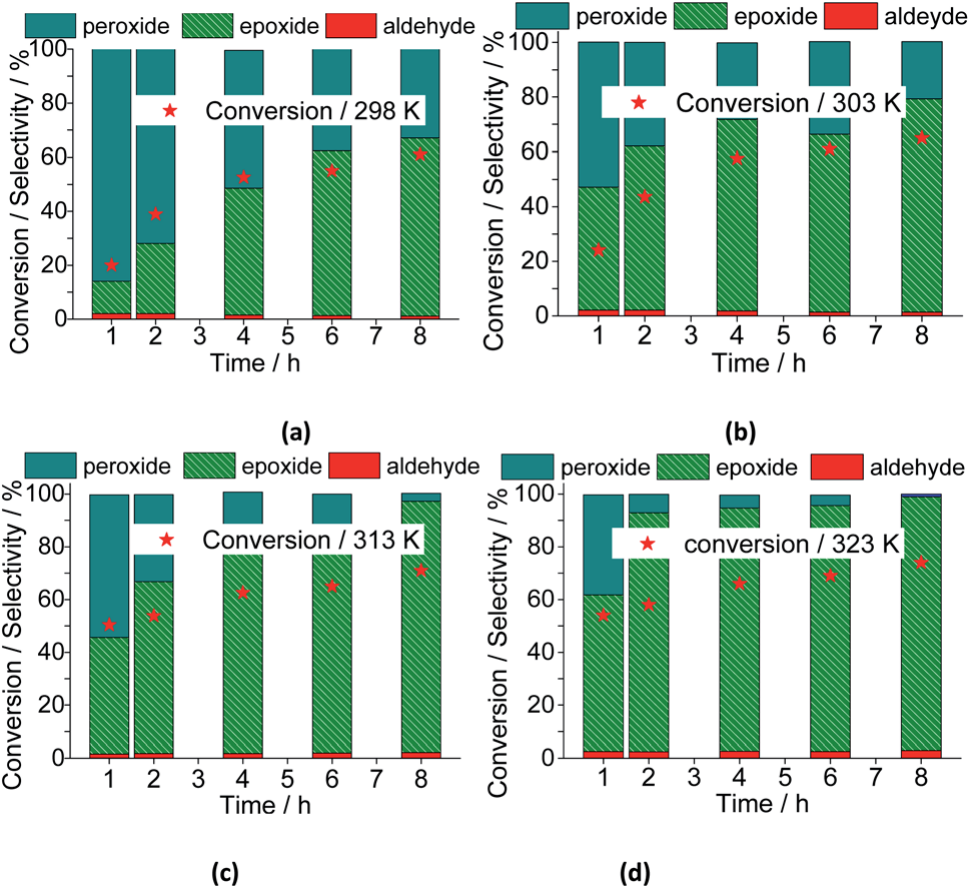
Effect of temperature on the conversion and reaction selectivity *versus* time of Na_4_PMo_11_VO_40_-catalyzed oxidation reactions of nerol with H_2_O_2_. Reaction conditions: Nerol (2.75 mmol), H_2_O_2_ (2.75 mmol), Na_4_PMo_11_VO_409_ (0.083 mol%), CH_3_CN (10 mL).

With a higher temperature the reactions became faster, due to the greater number of effective collisions, an effect that was much more visible at 333 K (Fig. 18). Previously, we have seen that the reactions carried out at 333 K achieved the conversion maximum within the first hour of reaction, regardless of the reaction conditions (*i.e.*, different stoichiometry, catalysts, *etc.*). Nonetheless, it was happening because the reactions have been performed with 0.66 mol% of catalyst load at 333 K. Herein, using the lowest catalyst load (*ca.* 0.083 mol%), only the reaction carried out at 333 K had a similar behavior; those other reactions performed at temperatures lower than 333 K, had their conversions gradually increasing with the run time (Fig. 19).

When we analyzed the behavior of the reaction selectivity through the time, we verified that at the temperature range of 298 to 323 K, an increase in temperature accelerated the reactions as well as the conversion of nerol peroxide to final oxidation products (*i.e.*, nerol epoxide, aldehyde and sometimes diepoxide) (Fig. 19). At the lowest temperature (*ca.* 333 K, Fig. 18a), the decomposition of peroxide intermediate to final products was compromised and after 8 h of reaction the lowest selectivity to nerol epoxide was achieved.

#### 3.2.7. Na_4_PMo_11_VO_40_-catalyzed oxidation reactions with H_2_O_2_: effect of the substrate

**Fig. 20:**
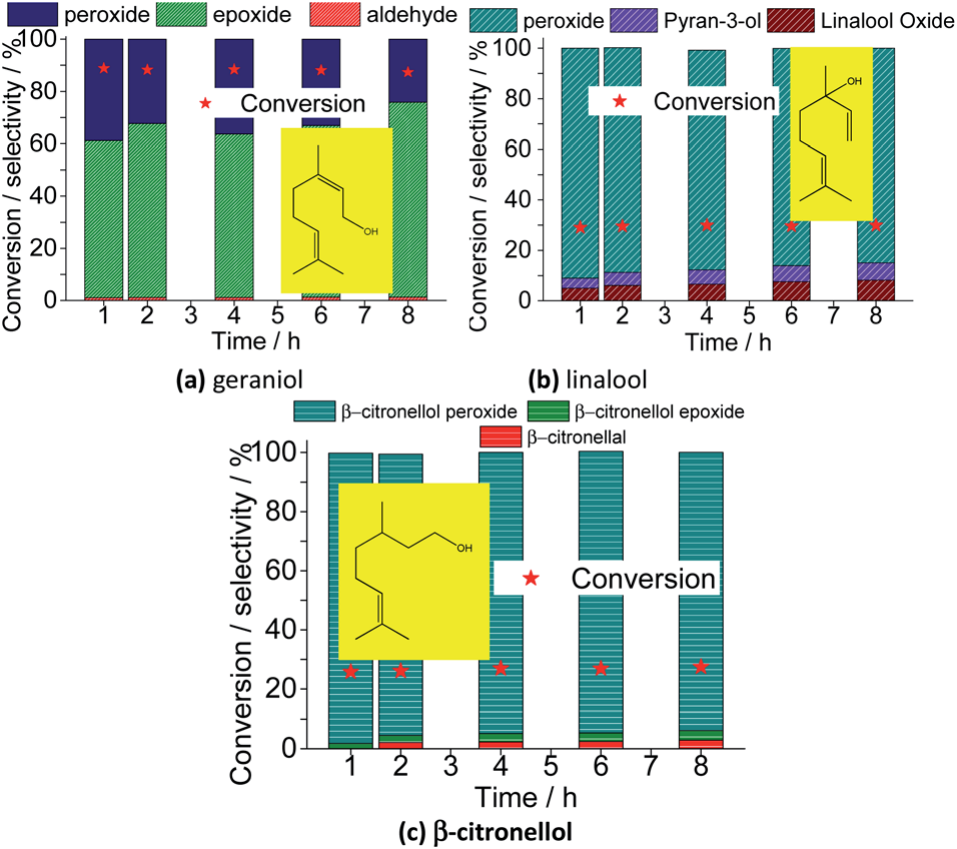
Effect of terpenic alcohols on the conversion of Na_4_PMo_11_VO_40_-catalyzed oxidation reactions with H_2_O_2_; (a) geraniol, (b) linalool and (c) citronellol. Reaction conditions: terpenic alcohol (2.75 mmol); H_2_O_2_ (2.75 mmol); Na_4_PMo_11_VO_40_ (0.66 mol%); temperature (333 K); CH_3_CN (10 mL).

To verify the possible electronic and steric effects on the reaction selectivity, we selected some terpenic alcohols with different structures: geraniol (Fig. 20a), a geometric isomer of nerol, linalool (Fig. 20b), a tertiary allylic alcohol, and β-citronellol (Fig. 20c), a primary alcohol. The variation of conversion and reaction selectivity *versus* time are shown in Fig. 20.

Besides nerol, all three terpenic alcohols (*i.e.*, geraniol, linalool, β-citronellol) with double bonds that may be potentially epoxidized were assessed. Geraniol and nerol are geometric isomers (*i.e.*, *E* and *Z* isomers, respectively), and showed a similar reactivity. It suggests that no stereochemistry controlling occur on the oxidative process of these two alcohols. Likewise verified in nerol oxidation, geraniol was quickly peroxidized and then converted to epoxide as the main product. Nonetheless, different than oxidation of nerol, where no peroxide was detected at the end of the reaction, a significant amount of the peroxidized substrate (*ca.* 20%) remained not converted after 8 h of reaction. Moreover, only a minimum portion was converted to aldehyde.

When a tertiary allylic alcohol was tested (*i.e.*, linalool, Fig. 20b), only a minimum formation of epoxide was noticed. The terminal double bond was less sensitivity to the epoxidation, an evidence that this is a hydroxy group-assisted reaction, being thus less favorable herein. The major product was peroxide linalool. A cyclization product of linalool was also formed, similarly to the verified in a previous work, where the lacunar sodium phosphotungstate was the catalyst.^35^

Finally, when primary alcohol without an allylic double bond was the substrate (β-citronellol, Fig. 20c), only a poor conversion was achieved, with a low selectivity toward aldehyde. Moreover, the trisubstituted double bond remained almost intact, likewise to the verified in the oxidation reactions of nerol or geraniol substrates.

## 4. Conclusions

Keggin-type heteropolyanions PMo_12−*n*_V_*n*_O_40_^(3+*n*)−^ (*n* = 0, 1, 2, or 3) in the form of sodium salts were synthesized and evaluated as catalysts in green oxidation routes of terpene alcohols with hydrogen peroxide. The effect of the main reaction parameters was assessed. Among the several sodium salts tested, the Na_4_PMo_11_VO_40_ was the most active and selective catalyst toward the formation of nerol epoxide. We have come up with a sustainable catalytic system for epoxidation reactions catalyzed by vanadium-doped phosphomolybdate, using a very low loading of catalyst (*ca.*, 0.032 mol%), achieving good-to-excellent yields (*ca.* 85–95%) of nerol epoxide selectivity, using a stoichiometric molar ratio of substrate oxidant (*ca.* 1 : 1). Temperature reaction, catalyst load, as well as the content of vanadium on the sodium phosphomolybdate were the key aspects that drive the efficiency of the process. The greatest activity of Na_4_PMo_11_VO_40_ catalyst was ascribed to electronic and acidity properties, as demonstrated by the UV-Vis, FT-IR, *n*-butylamine potentiometric titrations analyses. This catalyst system provides an efficient, convenient, and practical method for the synthesis of epoxides from allylic alcohols from renewable origin feedstock (*i.e.*, terpene alcohols), and a friendly oxidant (*i.e.*, hydrogen peroxide).

## Conflicts of interest

There are no conflicts to declare.

## Supplementary Material

RA-011-D1RA04191F-s001
