# Supplementary material for: Vanadium-doped sodium phosphomolybdate salts as catalysts in the terpene alcohols oxidation with hydrogen peroxide
Source: RSC Adv. 2021 Jul 8;11(39):24072–85. doi: 10.1039/d1ra04191f (PMC9036646; doi:10.1039/d1ra04191f)
Supplement: RA-011-D1RA04191F-s001 [file RA-011-D1RA04191F-s001.pdf]

Electronic Supplementary Material (ESI) for RSC Advances.
This journal is © The Royal Society of Chemistry 2021

Vanadium-doped Sodium phosphomolybdate salts as catalysts in the terpene alcohols oxidation with hydrogen peroxide

CASTELO BANDANE VILANCULO[1*], MÁRCIO JOSÉ DA SILVA[2], ALANA ALVES RODRIGUES, SUKARNO OLAVO FERREIRA[2] AND RENE CHAGAS DA SILVA[2]

[1]*Chemistry Department, Pedagogic University of Mozambique, FCNM, Campus de Lhanguene, Av. de Moçambique, km 1, Maputo, C.P.: 4040, Fax: (+258)21401082.*

[2]*Chemistry Department, Federal University of Viçosa, Viçosa, Minas Gerais, Brasil. zip-code: 36590-000*

Supplemental material

List of Figures

Figure 1SM. FT-IR spectra of Sodium phosphomolybdate and vanadophosphomolybdate Sodium salts.

Figure 2SM. UV-Vis spectra of Vanadium trisubstituted phosphomolybdate catalyst in aqueous solution.

Figure 3SM. Secondary structure of Na4PMo$_{11}$VO$_{40}$

Figure 4SM. Isotherms of adsorption and desorption of N_2 and volume and diameters porous (inset) of pure Sodium phosphomolybdate catalyst and after the Vanadium doping.

Fig. 5SM Scanning electronic microscopy images of undoped and Vanadium-doped Sodium phosphomolybdate salts.

Fig. 6SM EDS spectra of undoped and Vanadium-doped Sodium phosphomolybdate salts.

List of Tables

Table 1SM. Porosimetry characteristics of pure and Vanadium doped-Sodium phosphomolybdate salts[a]

Table 2SM. Hydration water number per mol of catalyst determined through thermal analysis.

Table 3SM. Elemental analysis data theoretical and experimental obtained from undoped and Vanadium-doped phosphomolybdate salts.

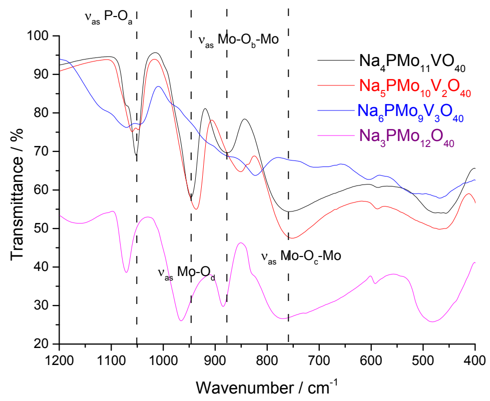

Fig. 1SM FT-IR spectra of Sodium phosphomolybdate salts unsubstituted and Vanadium substituted.

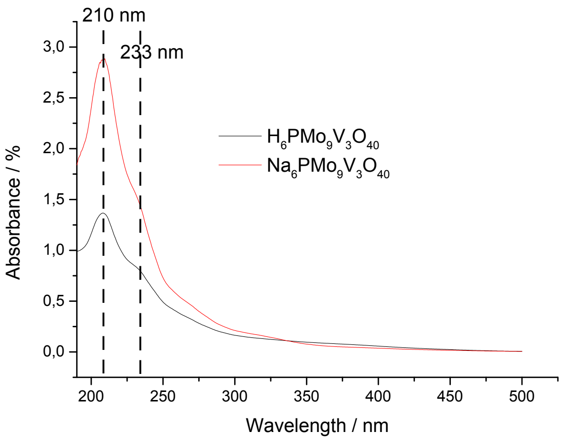

Figure 2SM. UV-Vis spectra of Vanadium trisubstituted phosphomolybdate catalyst in aqueous solution

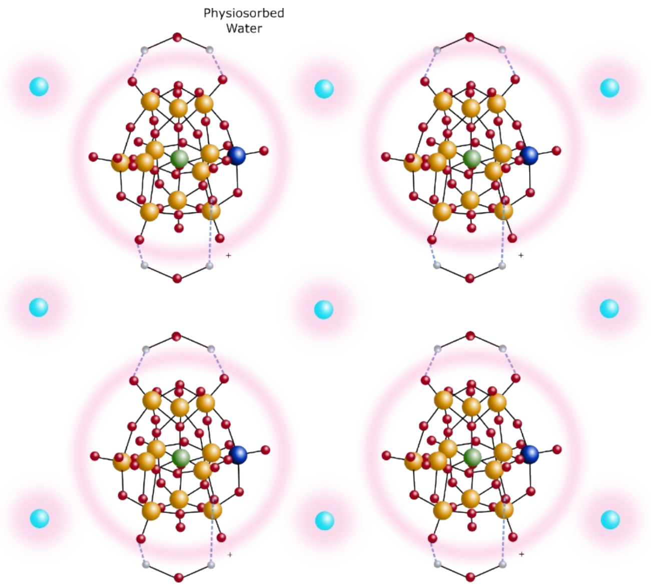

Fig. 3SM. Secondary structure of Na4P$Mo_{11}VO_{40}$

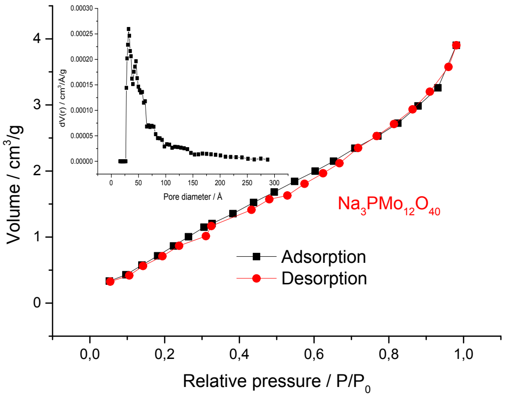

(a)

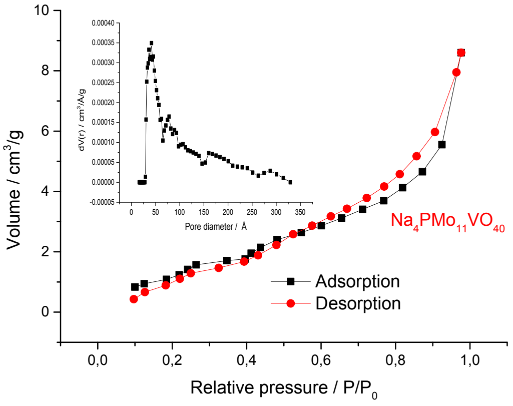

(b)

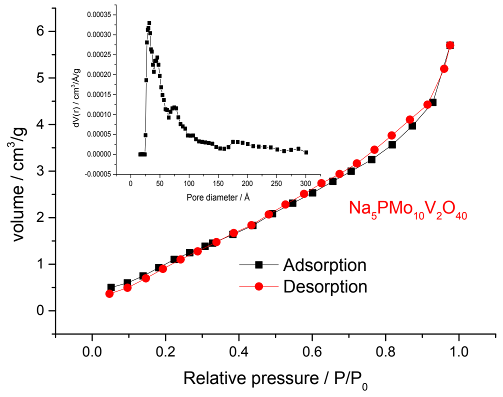

(c)

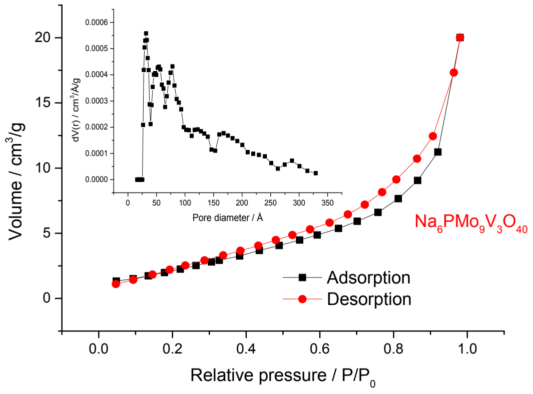

(d)

Fig. 4SM Isotherms of adsorption and desorption of N_2, volume, and distribution of porous diameters (inset) of pure Sodium phosphomolybdate catalyst before (a) and after the Vanadium doping (b-d).

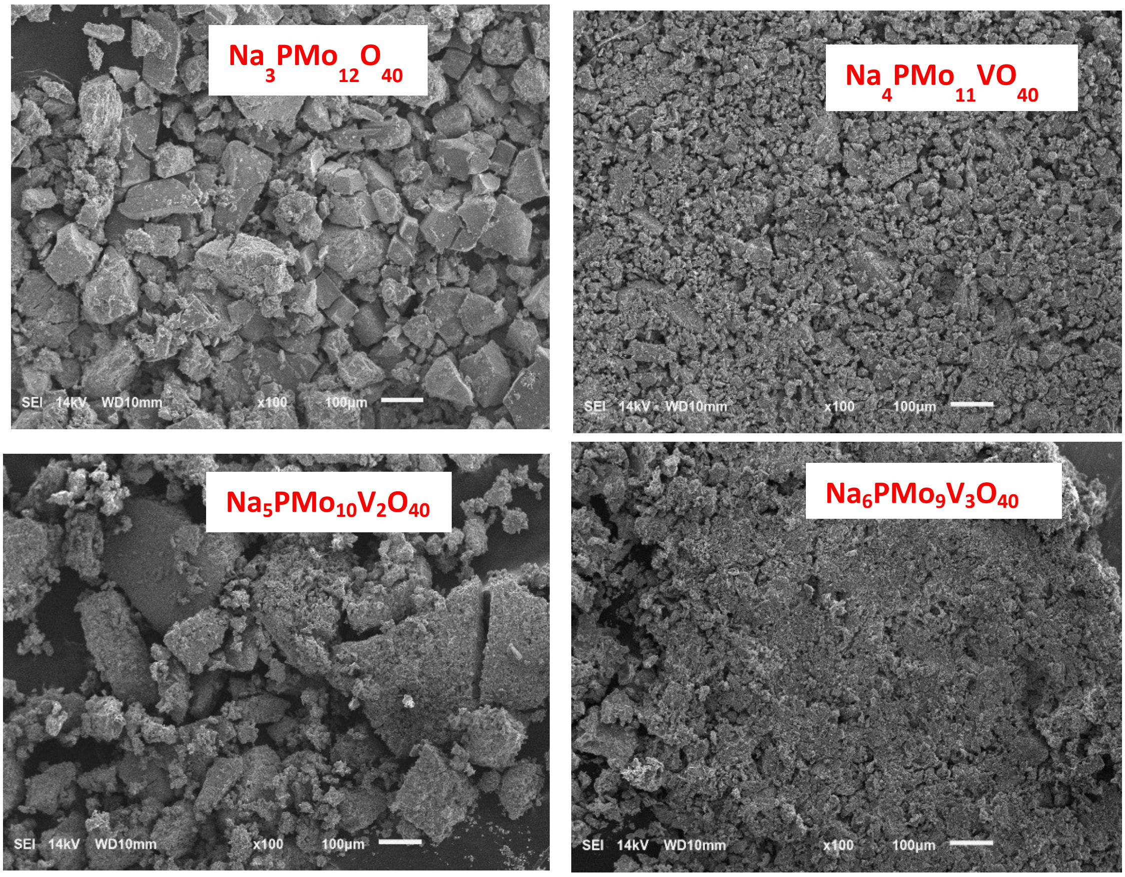

Fig. 5SM Scanning electronic microscopy images of undoped and Vanadium-doped Sodium phosphomolybdate salts.

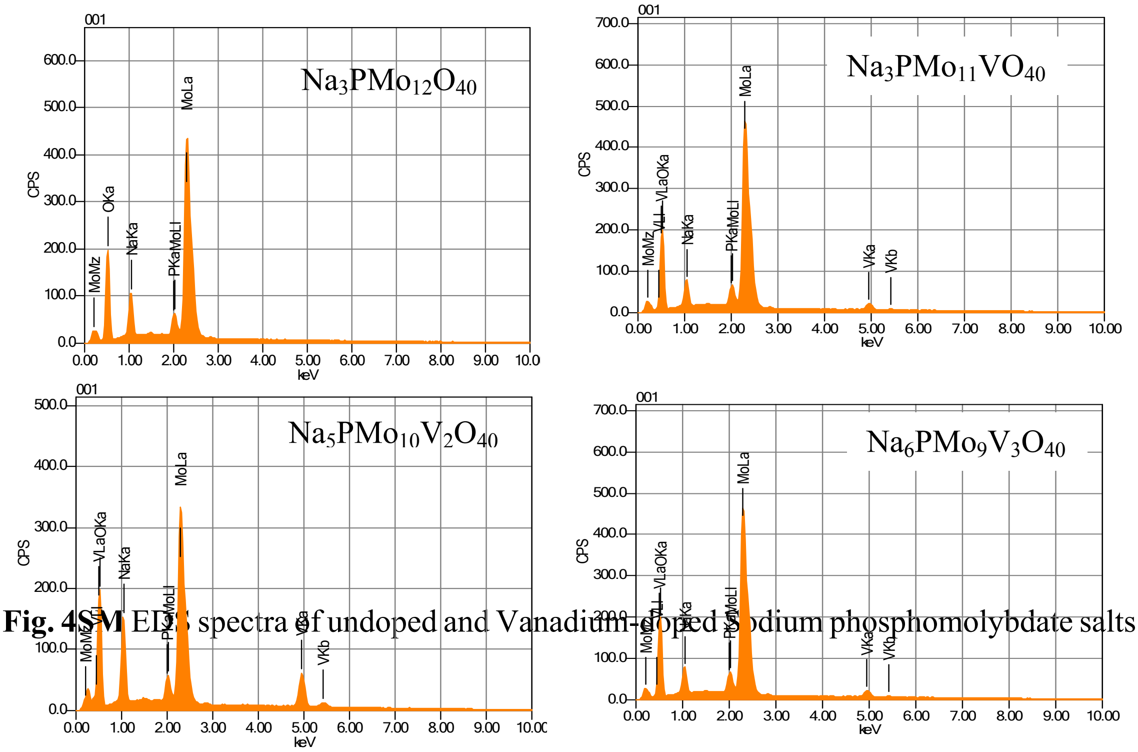

Fig. 4SM EDS spectra of undoped and Vanadium-doped Sodium phosphomolybdate salts

Fig. 6SM EDS spectra of undoped and Vanadium-doped Sodium phosphomolybdate salts.

Table 1SM. Porosimetry characteristics of undoped and Vanadium doped-Sodium phosphomolybdate salts[a]

Catalyst	S_{BET} (m^2/g)	V_{DFT} (cm^3/g)	D (Å)
$Na_3PMo_{12}O_{40}$	1.8	5.2 x 10^{-3}	31.7
$Na_4PMo_{11}VO_{40}$	3.1	1.5 x10^{-3}	41.5
$Na_5PMo_{10}V_2O_{40}$	3.7	7.6 x 10^{-3}	31.7
$Na_6PMo_9V_3O_{40}$	6.4	25.3 x 10^{-3}	31.7

[a]S_{BET} = surface area; V_{DFT} = cumulative pore volume; D = pore diameter

Table 2SM. Hydration water number per mol of catalyst determined through thermal analysis (TG/DTG).

Catalyst	Total hydration water (573 K)
$Na_3PMo_{12}O_{40}$	10
$Na_4PMo_{11}VO_{40}$	7
$Na_5PMo_{10}V_2O_{40}$	10
$Na_6PMo_9V_3O_{40}$	13

Table 3SM. Elemental analysis data theoretical and experimental obtained from undoped and Vanadium-doped phosphomolybdate salts.

Catalyst	Elemental composition (wt. %)									
	Na		P		Mo		V		O	
	Calc.	Exp.	Calc.	Exp.	Calc.	Exp.	Calc.	Exp.	Calc.	Exp.
$Na_3PMo_{12}O_{40}$	4	5	2	2	61	62	0	0	33	31
$Na_4PMo_{11}VO_{40}$	5	5	2	2	56	58	3	3	34	33
$Na_5PMo_{10}V_2O_{40}$	6	8	2	2	52	50	5	9	35	31
$Na_6PMo_9V_3O_{40}$	8	10	2	2	47	45	8	12	35	31
